# Ventral pallidal GABAergic neurons control wakefulness associated with motivation through the ventral tegmental pathway

**DOI:** 10.1038/s41380-020-00906-0

**Published:** 2020-10-14

**Authors:** Ya-Dong Li, Yan-Jia Luo, Wei Xu, Jing Ge, Yoan Cherasse, Yi-Qun Wang, Michael Lazarus, Wei-Min Qu, Zhi-Li Huang

**Affiliations:** 1 Department of Pharmacology, School of Basic Medical Sciences; State Key Laboratory of Medical Neurobiology and MOE Frontiers Center for Brain Science, and Institutes of Brain Science, Fudan University, Shanghai, 200032 China; 2 International Institute for Integrative Sleep Medicine (WPI-IIIS), University of Tsukuba, 1-1-1 Tennodai, Tsukuba, Ibaraki 305-8575 Japan

**Keywords:** Neuroscience, Psychology

## Abstract

The ventral pallidum (VP) regulates motivation, drug addiction, and several behaviors that rely on heightened arousal. However, the role and underlying neural circuits of the VP in the control of wakefulness remain poorly understood. In the present study, we sought to elucidate the specific role of VP GABAergic neurons in controlling sleep–wake behaviors in mice. Fiber photometry revealed that the population activity of VP GABAergic neurons was increased during physiological transitions from non-rapid eye movement (non-REM, NREM) sleep to either wakefulness or REM sleep. Moreover, chemogenetic and optogenetic manipulations were leveraged to investigate a potential causal role of VP GABAergic neurons in initiating and/or maintaining arousal. In vivo optogenetic stimulation of VP GABAergic neurons innervating the ventral tegmental area (VTA) strongly promoted arousal via disinhibition of VTA dopaminergic neurons. Functional in vitro mapping revealed that VP GABAergic neurons, in principle, inhibited VTA GABAergic neurons but also inhibited VTA dopaminergic neurons. In addition, optogenetic stimulation of terminals of VP GABAergic neurons revealed that they promoted arousal by innervating the lateral hypothalamus, but not the mediodorsal thalamus or lateral habenula. The increased wakefulness chemogenetically evoked by VP GABAergic neuronal activation was completely abolished by pretreatment with dopaminergic D_1_ and D_2_/D_3_ receptor antagonists. Furthermore, activation of VP GABAergic neurons increased exploration time in both the open-field and light–dark box tests but did not modulate depression-like behaviors or food intake. Finally, chemogenetic inhibition of VP GABAergic neurons decreased arousal. Taken together, our findings indicate that VP GABAergic neurons are essential for arousal related to motivation.

## Introduction

The ventral pallidum (VP) is a major component of the basal ganglia, which plays a key role in regulating locomotion, learning, and motivation, all of which rely on heightened arousal [1–4]. Functional disorders of the VP are associated with neuropsychiatric disorders [5], including depression [6] and drug addiction [7–9], which are often accompanied by sleep disturbances [5, 10], suggesting that the VP may play an important role in the regulation of sleep and wakefulness. However, whether the VP is involved in sleep–wake regulation remains poorly understood.

The VP receives dense inputs from the nucleus accumbens (NAc), which is mostly composed of GABAergic dopamine D_1_ receptor (D_1_R)-positive neurons in the direct pathway and D_2_R-positive neurons in the indirect pathway [11]. Our previous studies have shown that NAc D_1_R neurons and NAc D_2_R neurons promote arousal and sleep, respectively [12]. Both NAc D_1_R neurons and NAc D_2_R neurons send dense inputs to the VP; however, the precise role of the VP in sleep/wake regulation has not yet been elucidated. Blockade of GABA_A_ receptors in the VP via local administration of bicuculline, which activates VP neurons, greatly increases locomotion [13–15]. Other evidence shows that the VP promotes cocaine self-administration [16], which is accompanied by heightened arousal. Thus, we hypothesized that the VP represents a principal wake-promoting region.

The VP is a highly heterogeneous structure that is composed principally of GABAergic projection neurons [4]. Anatomical and functional studies have shown that VP GABAergic neurons regulate motivation, depression-like, and feeding behaviors via their projections to the mediodorsal thalamus (MD) [17], ventral tegmental area (VTA) [18, 19], lateral habenula (LHb) [6], and lateral hypothalamus (LH) [20]. However, it is unclear whether the VP also controls arousal through these neural circuits. Anatomical tracings and electrophysiological recordings have shown that VP GABAergic neurons send dense inputs to the VTA, and form connections with VTA neurons [21]. It is noteworthy that the VP drives positive reinforcement through the VTA [21], the latter of which is composed of wake-promoting dopaminergic and glutamatergic neurons, as well as arousal-reducing GABAergic neurons [22, 23]. Thus, the VTA represents a candidate region that receives direct axonal connections from VP GABAergic neurons. However, the putative circuit mechanism of the VP-VTA in sleep–wake regulation remains to be elucidated.

In the present study, we sought to uncover the specific role of VP GABAergic neurons in controlling sleep–wake behaviors. Fiber photometry unveiled arousal-correlated neuronal activity in VP GABAergic neurons across the spontaneous sleep–wake cycle. By using chemogenetic and optogenetic manipulations combined with polysomnographic recordings, we demonstrated that VP GABAergic neurons were sufficient to initiate and maintain behavioral arousal. In vivo and in vitro optogenetic-mediated circuit mapping revealed that VP GABAergic neurons regulated arousal through disinhibition of VTA dopaminergic neurons. Pharmacological manipulations further demonstrated that dopaminergic signaling was necessary for VP GABAergic neurons in promoting arousal. Importantly, we found that consistent with increased wakefulness, activation of VP GABAergic neurons concomitantly increased motivation, as demonstrated by longer exploration times. Finally, chemogenetic inhibition or lesioning of VP GABAergic neurons demonstrated that VP GABAergic neuronal activity was necessary for maintaining arousal. Collectively, our results provide several lines of evidence regarding VP GABAergic neurons and their circuit mechanisms in promoting arousal and motivation.

## Results

### **Population activity of VP GABAergic neurons is increased during non-rapid eye movement (NREM) sleep-to-wake transitions**

**Fig. 1:**
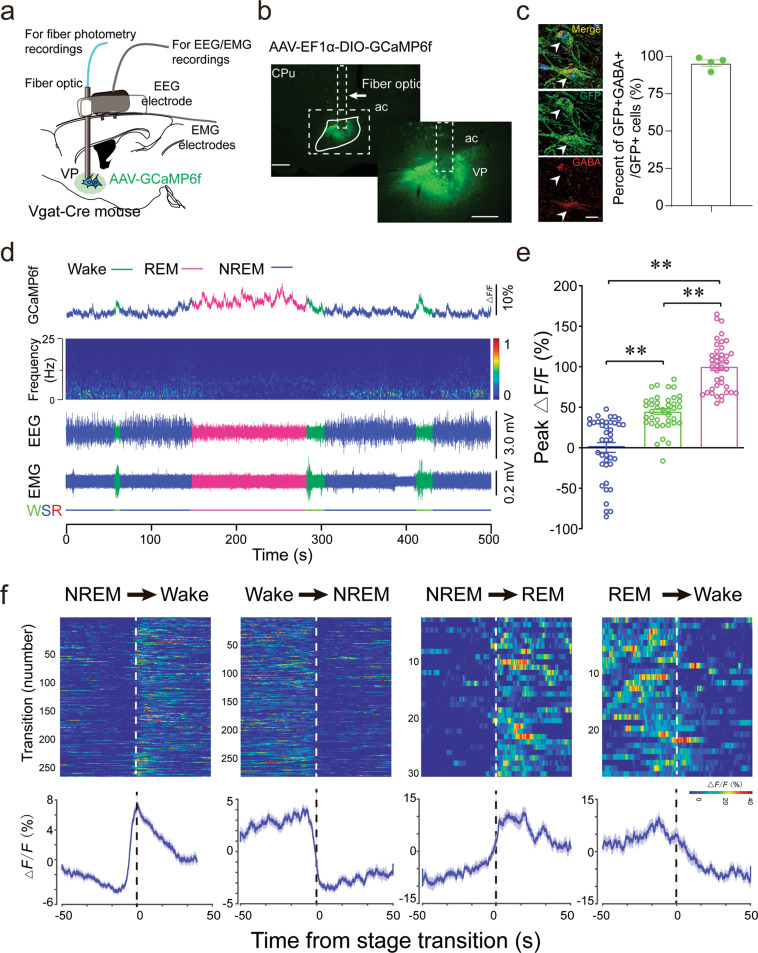
Population activity of VP GABAergic neurons across sleep–wake states. **a** Schematic of in vivo fiber-photometry recordings. **b** Unilateral viral targeting of AAV-EF1α-DIO-GCaMP6f into the VP of a Vgat-Cre mouse. Right: Viral expression of GCaMP6f and the placement of the fiber-optic probe in the VP. Scale bar = 200 μm. **c** GFP-expressing cells were GABA-positive. Scale bar = 20 μm (*n* = 4 mice). **d** Representative fluorescent traces, relative EEG power, and EEG/EMG traces across spontaneous sleep–wake states. **e** ∆*F/F* peaks during wakefulness, NREM sleep, and REM sleep. The fluorescent peak values were normalized by the mean ∆*F*/*F* peaks during NREM sleep (*n* = 4 mice, ten sessions per mouse, **P < 0.01, one-way ANOVA followed by Tukey’s post-hoc test). **f** Fluorescent signals aligned to arousal-state transitions. Upper panel: Individual transitions with color-coded fluorescent intensities. Lower panel: Average calcium transients from all the transitions, expressed as the mean ± SEM.

To investigate the physiological real-time activity of VP GABAergic neurons across spontaneous sleep–wake cycles in mice, we recorded calcium activity of VP GABAergic neurons via fiber photometry [24, 25]. The AAV_2_-Ef1α-DIO-GCaMP6f construct was unilaterally injected into the VP of Vgat-Cre mice, and an optical fiber was implanted into the VP. GCaMP6f signals and electroencephalograms (EEGs)/electromyograms (EMGs) were simultaneously recorded in freely moving mice within their home cages (Fig. 1a). We used immunofluorescent staining of substance P to label the boundary of the VP [4] (Supplementary Fig. 1a). GCaMP6f-expressing cells were located in the dorsolateral VP (Fig. 1b), and 95% of these cells overlapped with anti-GABA immunostaining (Fig. 1c). Interestingly, we observed that changes in the population activity of VP GABAergic neurons were highly and consistently associated with sleep–wake-stage transitions (Fig. 1d, e). In particular, VP GABAergic neurons displayed lower GCaMP6f activity during non-rapid eye movement (non-REM, NREM) sleep (i.e., high delta power density, increased EEG amplitude and decreased EMG activity), whereas they exhibited moderate activity during wakefulness (i.e., low delta power density, decreased EEG amplitude and increased EMG activity) and even higher activity during REM sleep (i.e., high theta power density, decreased EEG amplitude and no EMG activity) (Fig. 1d, e). Notably, a dramatic increase in the GCaMP6f activity of VP GABAergic neurons was initiated at ~4 s before NREM-to-wake and 2 s before NREM-to-REM transitions, whereas decreased GCaMP6f activity occurred before wake-to-NREM transitions or REM-to-wake transitions (Fig. 1f). Although the average calcium activity of VP GABAergic neurons decreased with prolonged time spent in wakefulness, the mean ∆*F*/*F* during wakefulness was higher than that during NREM sleep, indicating a potential role of VP GABAergic neurons in maintaining arousal. These findings demonstrate that the population activity of VP GABAergic neurons was associated with sleep–wake transitions. Higher activity of VP GABAergic neurons was appeared in transitions from NREM sleep to wakefulness, suggesting a physiological role of VP GABAergic neurons in the regulation of wakefulness.

### Chemogenetic or optogenetic activation of VP GABAergic neurons is sufficient to induce and maintain wakefulness

**Fig. 2:**
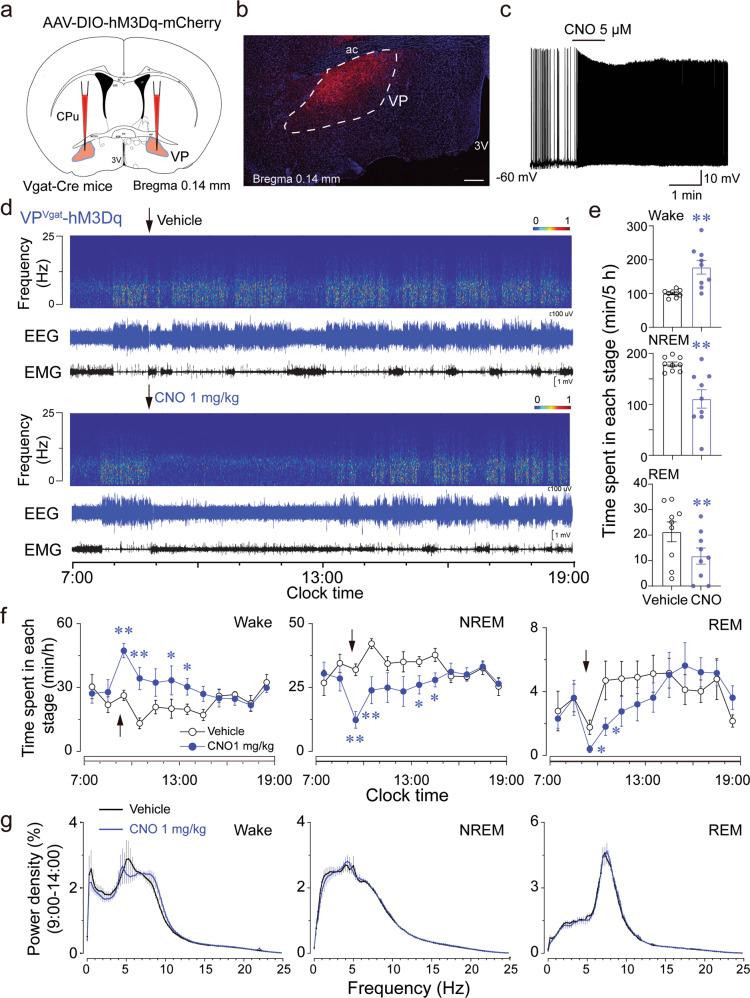
Chemogenetic activation of VP GABAergic neurons promotes arousal. **a** Schematic diagram of chemogenetic activation of VP GABAergic neurons. **b** The VP was infected by the hM3Dq-mCherry virus. Scale bar = 200 µm. **c** Bath application of CNO at 5 µM increased firings in an hM3Dq-mCherry neuron. **d** Typical examples of a relative EEG power heatmap and EEG/EMG traces after vehicle or CNO (1 mg/kg) injection at 9:00 in an hM3Dq Vgat-Cre mouse. **e** The total time spent in NREM sleep and wakefulness for 5 h after administration of vehicle or CNO. **f** Time-course of wakefulness, NREM sleep, and REM sleep in Vgat-Cre mice expressing hM3Dq after administration of vehicle or CNO. **g** EEG power density for 5 h after vehicle or CNO administration. Data shown are the mean ± SEM (*n* = 9; *P < 0.05, **P < 0.01, using repeated-measures ANOVA followed by paired  *t*-test).

Having identified increased activity of VP GABAergic neurons during transitions from NREM sleep to wakefulness, we next interrogated the causal role of VP GABAergic neurons in regulating arousal. Chemogenetic and optogenetic approaches were used to manipulate the activity of VP GABAergic neurons during simultaneous EEG/EMG recordings. The AAV_10_-hSyn-DIO-hM3Dq-mCherry construct was bilaterally injected into the VP of Vgat-Cre mice (Fig. 2a). Immunochemical staining showed that mCherry was precisely expressed in the VP (Fig. 2b, Supplementary Fig. 1b–e). In vitro electrophysiological experiments showed that hM3Dq+ GABAergic neurons were activated after clozapine-n-oxide (CNO) application (Fig. 2c). C-Fos staining confirmed the activation of mCherry+ neurons by CNO injection (Supplementary Fig. 2a–c). Importantly, chemogenetic activation of VP GABAergic neurons significantly increased wakefulness and concomitantly decreased both NREM and REM sleep during the 5 h following CNO (1 mg/kg) administration at 09:00 (light on at 07:00), as compared with these parameters in the vehicle control (Fig. 2d–f). CNO administration induced a decrease in low frequency, high-amplitude EEG activity together with an increase in EMG activity (Fig. 2d), suggesting that chemogenetic activation of VP GABAergic neurons induced behavioral arousal. Administration of CNO at 1 mg/kg resulted in a 78% increase in wakefulness and a 38% reduction in NREM sleep during the 5-h post-injection period (Fig. 2e, f). In contrast, there was no significant difference in the EEG power density at each stage (Fig. 2g), further indicating that activation of VP GABAergic neurons induced physiological arousal.

Next, we used optogenetics to precisely activate VP GABAergic neurons and to clarify whether activation of VP GABAergic neurons could initiate wakefulness. The AAV_10_-hSyn-DIO-ChR2-mCherry construct was injected into the bilateral VP with optical fibers targeting these neurons in Vgat-Cre mice (Supplementary Fig. 3a, b). Optogenetic stimulation increased c-Fos expression in mCherry+ neurons (Supplementary Fig. 3c). Acute optogenetic activation of VP GABAergic neurons awakened mice from NREM sleep, with a significant increase in EMG activity after EEG desynchronization (Supplementary Fig. 3d). Short latencies for sleep-to-wake transitions were observed during blue-light pulses at frequencies from 5–30 Hz (Supplementary Fig. 3e). Optogenetic stimulation induced wakefulness during periods of NREM sleep in all trials (35 trails) in VP-ChR2 mice, versus only 4 out of 40 trials in VP-mCherry control mice (Supplementary Fig. 3f). Importantly, chronic optogenetic stimulation (5-ms pulses at 20 Hz, with 10-s on/20-s off for 120 cycles) of VP GABAergic neurons for 1 h consistently maintained long-term arousal in VP-ChR2 mice (Supplementary Fig. 3g, h). Furthermore, the time spent in arousal was increased by 2.1-fold, with a 97% decrease in NREM sleep. Taken together, these findings indicate that chemogenetic or optogenetic activation of VP GABAergic neurons was sufficient to initiate and maintain behavioral arousal.

### VP GABAergic neurons regulate arousal through the VTA pathway

**Fig. 3:**
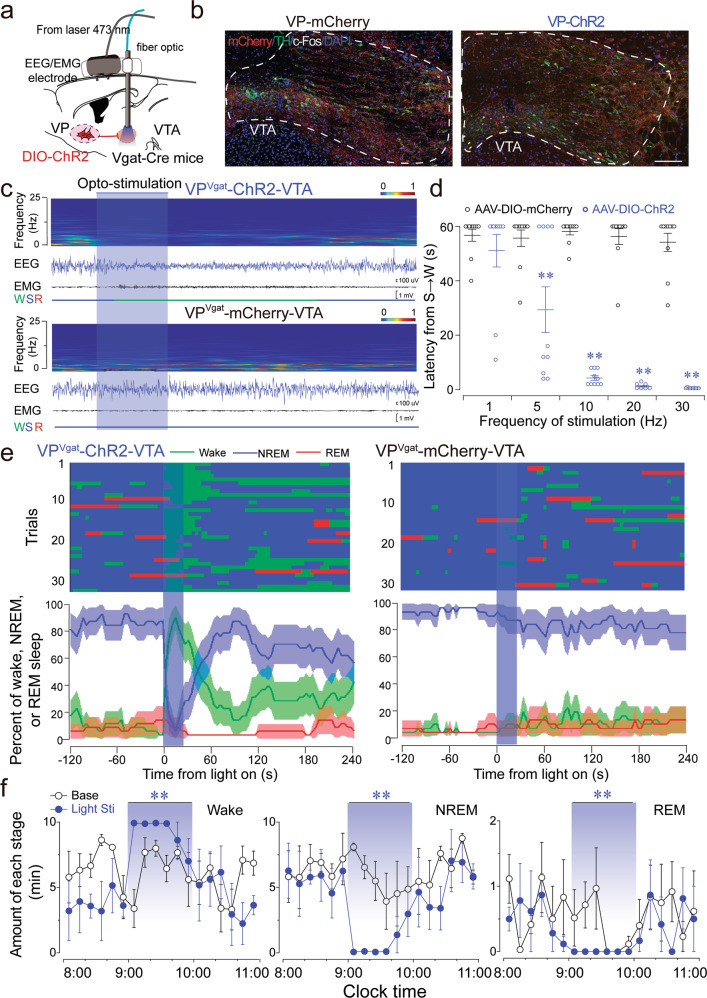
Optogenetic stimulation of the VP^GABA^-VTA pathway induces arousal. **a** Sagittal diagram for in vivo optogenetic stimulation of the VP^GABA^-VTA pathway in Vgat-Cre mice. **b** Optogenetic stimulation of axonal terminals of VP GABAergic neurons in the VTA induced dense c-Fos expression in TH+ neurons. **c** Optogenetic activation of the VP^GABA^-VTA pathway induced an immediate transition from NREM sleep to wakefulness. The EEG amplitude was decreased, with a decreasing delta power and increasing EMG activity, after blue-light stimulation in ChR2 mice, but not in mCherry control mice. **d** The latency from NREM sleep to wakefulness decreased with increased stimulation frequency. **e** Sleep stages after VTA blue-light stimulation in ChR2-mCherry mice or mCherry control mice. **f** Durations of wakefulness, NREM sleep, and REM sleep during 1-h optogenetic stimulation of the VP^GABA^-VTA pathway during the light period (*n* = 4. **P < 0.01, using repeated-measures ANOVA, followed by paired *t*-test).

To uncover the circuit mechanism of VP GABAergic neurons in regulating arousal, we first performed anterograde tracing of VP GABAergic neurons (Supplementary Fig. 2d–l). AAV-DIO-mCherry was injected into the VP, after which abundant mCherry terminals were found in the VTA (Supplementary Fig. 2e–h), which is consistent with previous findings that the VP innervates VTA neurons [6, 21]. Surprisingly, after chemogenetic activation of VP GABAergic neurons, we found a robust increase of c-Fos expression in VTA tyrosine hydroxylase-positive (TH+) neurons (Supplementary Fig. 2m, p), which play an important dopaminergic role in the regulation of arousal and motivation-related behaviors [22]. Next, we employed an optogenetic approach to precisely stimulate VP^GABA^-VTA projections in vivo and in vitro. The AAV_10_-hSyn-DIO-ChR2-mCherry construct was injected into the VP with bilateral optical fibers targeting axonal terminals in the VTA of Vgat-Cre mice (Fig. 3a). Blue-light stimulation induced intense c-Fos expression in the VTA (Fig. 3b), principally in TH+ neurons (Supplementary Fig. 4a, b), suggesting that optogenetic stimulation of VP^GABA^-VTA projections in vivo activated VTA dopaminergic neurons. Optogenetic stimulation of the VTA at 20 Hz immediately awakened mice from NREM sleep, inducing decreased EEG delta power/amplitude, while concomitantly increasing EMG activities (Fig. 3c), suggesting that stimulation of VP^GABA^-VTA projections induced behavioral arousal. Short latencies for sleep-to-wake transitions were observed during blue-light pulses onto the VTA at frequencies ranging from 5–30 Hz (Fig. 3d). In total, we performed 33 trials of optogenetic stimulation in VP-ChR2-VTA mice and found that mice awakened from NREM sleep in 30 trials, versus only 2 of 31 trials in VP-mCherry-VTA control mice (Fig. 3e). In addition, chronic stimulation of VP^GABA^-VTA projections for 1 h also dramatically increased long-term arousal (Fig. 3f). These data reveal that the activation of VP GABAergic projections to the VTA was sufficient to promote arousal.

### VP GABAergic neurons form monosynaptic connections with both VTA dopaminergic and GABAergic neurons

VP GABAergic neurons send inhibitory inputs to the VTA [6, 26]; however, chemogenetic activation of VP GABAergic neurons and optogenetic activation of VP^GABA^-VTA projections in vivo increased VTA TH+ neuronal activity, as demonstrated by c-Fos staining. In order to further explore the circuit mechanism of VP GABAergic neurons in regulating arousal, we injected the AAV_10_-hSyn-DIO-ChR2-mCherry construct into the VP of Vgat-Cre:GAD67-GFP double-transgenic mice and subsequently recorded activity of VTA neurons under blue-light stimulation in acute slices in vitro (Supplementary Fig. 5a–d). We identified TH+ neurons, TH-negative (TH−) GABAergic neurons, and TH− glutamatergic neurons via single-cell RT-PCR (Supplementary Fig. 5e), electrophysiological characteristics (Supplementary Fig. 5f, j), and GFP fluorescence (Supplementary Fig. 5g, k). Interestingly, VP GABAergic neurons formed direct connections with both VTA TH+ neurons and GABAergic TH− neurons, through which VP GABAergic neurons sometimes inhibited wake-promoting TH+ neurons but predominantly inhibited TH− neurons (18/21 in TH− cells *vs* 21/37 in TH+ cells, *P* < 0.05, Supplementary Fig. 5b). Although the latencies and amplitudes of light-induced inhibitory postsynaptic currents (IPSCs) in TH+ and TH− cells were not significantly different from one another, the latencies of IPSCs from 6/21 connected TH+ cells were longer than 5 ms, indicating that VP GABAergic neurons may also indirectly innervate these TH+ cells, while the latencies of all connected TH− cells were less than 5 ms, suggesting that the latter were all monosynaptic connections (Supplementary Fig. 5c, d). Single-cell PCR showed that the majority of GFP-negative cells (non-GABAergic cells, 6 out of 7 cells) expressed TH RNA, whereas only one of these cells expressed vesicle glutamate transporter 2 (Vglut2) RNA, indicating that most GFP-negative cells were TH+ dopaminergic neurons (Supplementary Fig. 5e). Under the cell-attached mode, optogenetic stimulation of axonal terminals from VP GABAergic neurons in the VTA inhibited the firing of both TH+ cells (Supplementary Fig. 5f–i) and TH− cells (Supplementary Fig. 5j–m) via light-evoked IPSCs, which were blocked by the GABA_A_ receptor antagonist, gabazine (SR-95531) (Supplementary Fig. 5i, m). In addition, we found that three TH+ cells, but no TH− cells, were inhibited by VP GABAergic inputs through GABA_B_ receptors (Supplementary Fig. 6a–e). Taken together, our c-Fos and electrophysiological results demonstrate that VP GABAergic neurons principally targeted VTA TH− cells, resulting in disinhibition of TH+ cells, which putatively underlies control of arousal by VP GABAergic neurons.

### VP GABAergic neurons regulate arousal partially through the LH pathway

Our anterograde tracing data showed that VP GABAergic neurons projected to the LH (Supplementary Fig. 2i–l), the latter of which plays an important role in sleep–wake regulation [27]. Chemogenetic activation of VP GABAergic neurons increased c-Fos expression in LH orexinergic neurons (Supplementary Fig. 2n, q) but not in melanin-concentrating hormone (MCH) neurons [28] (Supplementary Fig. 2o, r), indicating that VP GABAergic neurons may also regulate arousal through the LH pathway. Thus, we used an optogenetic approach to stimulate VP^GABA^-LH projections in vivo and in vitro (Supplementary Fig. 7a). Dense mCherry-positive axonal terminals of VP GABAergic neurons were found in the LH (Supplementary Fig. 7b), and optogenetic stimulation induced GABA_A_R-mediated IPSCs (Supplementary Fig. 7c–e). Subsequently, we stimulated VP^GABA^-LH projections in vivo (Supplementary Fig. 7f). Interestingly, optogenetic stimulation of VP^GABA^-LH projections also increased c-Fos expression in the LH (Supplementary Fig. 7g–h), indicating that several subtypes of LH neurons were disinhibited. In vivo optogenetic stimulation of VP^GABA^-LH projections also induced behavioral arousal (Supplementary Fig. 7i). Short latencies for sleep-to-wake transitions were observed during blue-light stimulation in the LH at frequencies ranging from 5–20 Hz (Supplementary Fig. 7j). In addition, chronic stimulation of VP^GABA^-LH projections for 1 h induced a 1.7-fold increase in wakefulness (Supplementary Fig. 7k, l). These data suggest that the LH was also an important downstream nucleus for VP GABAergic neurons in promoting arousal.

The MD and LHb are also innervated by VP GABAergic neurons. Hence, we also stimulated VP^GABA^-MD (Supplementary Fig. 8a–d) and VP^GABA^-LHb (Supplementary Fig. 8e–h) projections in vivo. However, optogenetic stimulation of these pathways did not alter sleep–wake transitions, suggesting that the MD and LHb were not involved in VP GABAergic neurons promoting arousal.

### Dopaminergic release is necessary for VP GABAergic neurons in regulating arousal

**Fig. 4:**
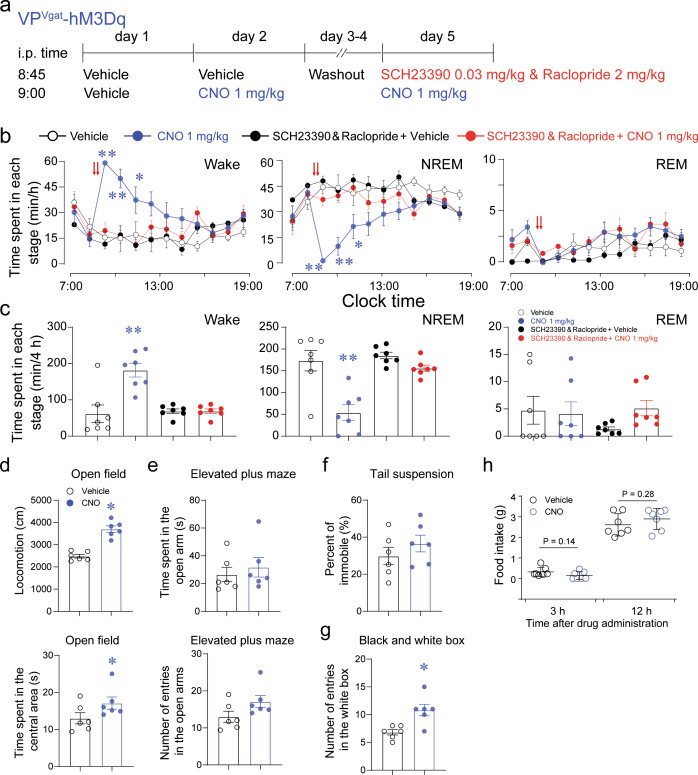
VP GABAergic neurons regulating arousal depend on dopaminergic signaling and induce motivation-related behaviors. **a** Schematic of pretreatment of dopaminergic receptor antagonists in VP^Vgat^-hM3Dq mice. **b** Time spent in wakefulness, NREM sleep, and REM sleep in Vgat-Cre mice after vehicle and vehicle, vehicle and CNO (1 mg/kg), or SCH23390 (0.03 mg/kg) and raclopride (2 mg/kg, i.p.) during concurrent CNO (1 mg/kg) administration. **c** The total amount of time spent in wakefulness, NREM sleep, and REM sleep from 9:00 to 13:00 in Vgat-Cre mice (*n* = 7 mice, *P < 0.05, **P < 0.01, using repeated-measures ANOVA, followed by Tukey post-hoc test). **d** Chemogenetic activation of VP GABAergic neurons increased locomotion and the time spent in the central area in the open-field test. **e**, **f** Activation of VP GABAergic neurons did not affect the time spent in open arms or the number of entries in open arms in the elevated plus-maze test (**e**) or the percent of immobility in the tail suspension test (**f**) after CNO administration. **g** The number of entries in the light box was increased after CNO administration in the light–dark box test (*n* = 6 mice, *P < 0.05, paired *t*-test). **h** Food intake within 3 h and 12 h after chemogenetic activation of VP GABAergic neurons in Vgat-Cre mice (*n* = 7 mice, paired *t*-test).

Our results showed that VP GABAergic neurons promoted arousal through the VTA within the ventral midbrain. To further investigate the role of dopamine in these effects, we investigated whether systemic blocking of D_1_ and D_2_ receptors would abolish wakefulness induced by chemogenetic activation of VP GABAergic neurons (Fig. 4a). We administered pretreatments of the dopaminergic D_1_ receptor antagonist, SCH23390 (0.03 mg/kg), and the dopaminergic D_2_/D_3_ receptor antagonist, raclopride (2 mg/kg), based on previous studies [29, 30], in Vgat-Cre mice that expressed hM3Dq in the VP. Chemogenetic activation of VP GABAergic neurons significantly increased wakefulness; however, this increased wakefulness was completely abolished by pretreatments with SCH23390 and raclopride (Fig. 4b, c). Treatment with SCH23390 and raclopride but without CNO did not increase arousal or NREM sleep in the light phase. These results further demonstrate that activation of VTA TH+ dopaminergic neurons plays a crucial role in mediating arousal controlled by VP GABAergic neurons.

### Activation of VP GABAergic neurons increases motivation

The VP has been reported to be involved in numerous motivation-related behaviors, including motor function, learning/memory, and reward [4]. In order to explore whether activation of VP GABAergic neurons promotes motivation-related arousal, we tested a series of behaviors after chemogenetic activation of VP GABAergic neurons. Chemogenetic activation of VP GABAergic neurons increased locomotion and the time spent in the central area in an open-field test (OFT) (Fig. 4d), indicating increased exploratory behavior. In contrast, there were no changes in behavior in the elevated plus-maze (EPM) test or the tail-suspension test (Fig. 4e, f), indicating that activation of VP GABAergic neurons did not change anxiety-like or depression-like behaviors. Importantly, activation of VP GABAergic neurons increased the number of entries into the light box during the light–dark box test, further indicating that activation of VP GABAergic neurons specifically promoted exploratory behavior (Fig. 4g). In addition, food intake within 3 h and 12 h after CNO administration was not significantly altered, indicating that activation of VP GABAergic neurons did not directly affect food intake (Fig. 4h). Taken together, increased arousal mediated by activation of VP GABAergic neurons was related to motivation-related exploratory behaviors.

### VP GABAergic neurons are essential for arousal

**Fig. 5:**
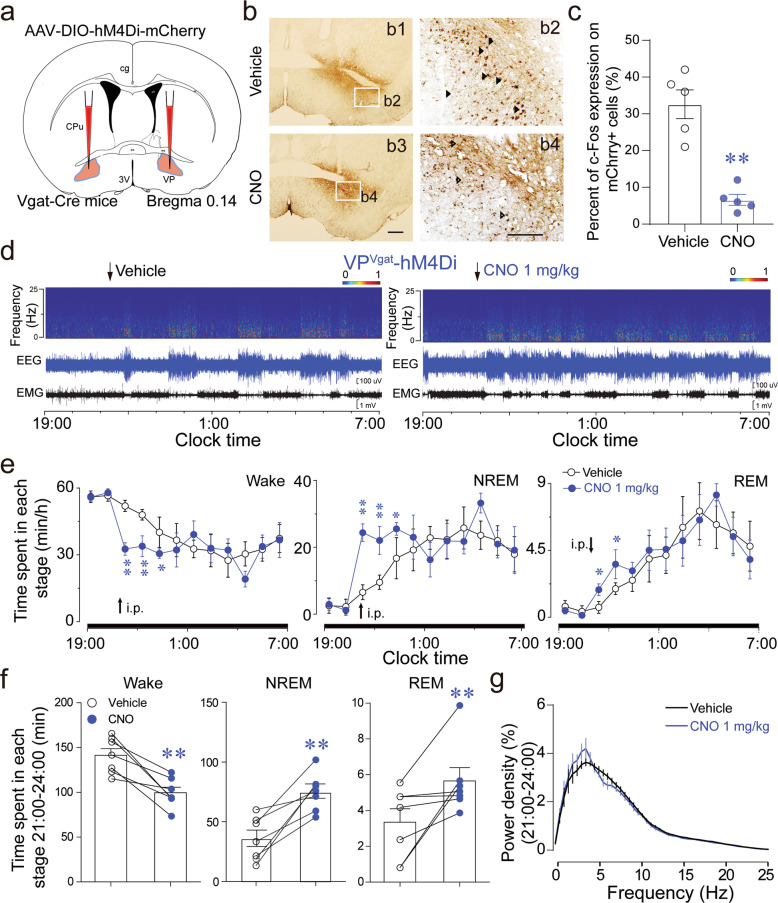
Chemogenetic inhibition of VP GABAergic neurons decreases the duration of wakefulness. **a** Schematic diagram of chemogenetic inhibition of VP GABAergic neurons. **b** c-Fos expression was decreased in the VP after CNO injection in hM4Di mice. Scale bars = 100 µm. **c** CNO administration decreased c-Fos expression in mCherry+ neurons by 74%. **d** Typical examples of relative EEG power and EEG/EMG traces after vehicle or CNO administration at 21:00 in an hM4Di Vgat-Cre mouse. **e** The time-course of Vgat-Cre mice expressing hM4Di after administration of vehicle or CNO. **f** The cumulative time of wakefulness, NREM sleep, and REM sleep after vehicle or CNO injection for 3 h. **g** EEG power density of NREM sleep for 3 h after vehicle or CNO administration (*n* = 7 mice per group). Data shown are the mean ± SEM (*P < 0.05, **P < 0.01, compared with vehicle control, two-way ANOVA followed by Tukey post-hoc test, paired or unpaired *t* test).

In order to determine whether the activity of VP GABAergic neurons is essential for sustaining arousal, we specifically injected the AAV_10_-hSyn-DIO-hM4Di-mCherry construct into the VP of Vgat-Cre mice (Fig. 5a). CNO administration inhibited VP GABAergic neurons, with a decrease in c-Fos expression within mCherry+ cells (Fig. 5b, c). Strikingly, the total duration of wakefulness was significantly decreased by 30% during 3 h after CNO administration at 21:00 (light off at 19:00), concomitant with a 1.1-fold increase in NREM sleep and a 65% increase in REM sleep, as compared with these parameters in vehicle controls (Fig. 5d–f). In addition, there was no significant difference in the power density of NREM sleep after CNO administration (Fig. 5g), indicating that inhibition of VP GABAergic neurons induced physiological sleep.

In addition, lesioning of VP GABAergic neurons decreased arousal in the dark phase (Supplementary Fig. 9). A combination of AAV_8_-hSyn-DIO-Caspase3 and AAV_8_-hSyn-DIO-eYFP constructs was bilaterally injected into the VP (Supplementary Fig. 9a), and ablation of GABAergic neurons was confirmed by anti-GABA immunofluorescent staining (Supplementary Fig. 9b). Consistent with our inhibition results, lesioning of VP GABAergic neurons decreased the time spent in wakefulness of the first 4 h in the dark phase (Supplementary Fig. 9c, d), as compared with that of GFP controls, whereas the EEG power density during NREM sleep was not altered (Supplementary Fig. 9e). Collectively, these results demonstrate that inhibition or lesioning of VP GABAergic neurons decreased the amount of wakefulness.

## Discussion

The VP is an important component of the ventral basal ganglia and regulates numerous motivation-related behaviors—including reward, motor function, and learning/memory—that rely on heightened arousal [2, 4, 31, 32]. In addition, the VP is connected to several sleep–wake regulatory nuclei, as it receives NAc projections and projects to both the VTA and LH [4, 33]. In the present study, we report that VP GABAergic neurons regulated behavioral arousal. A physiological correlation of VP GABAergic neurons and arousal was indicated by increased population activity during wakefulness. Furthermore, bidirectional chemogenetic and optogenetic manipulations demonstrated that VP GABAergic neurons were vital for promoting arousal. Moreover, we elucidated that VP GABAergic neurons regulated arousal through the VTA pathway, in which dopaminergic signaling was found to be necessary for arousal. Taken together, our findings identify a novel region in sleep–wake regulation and bridge several gaps in the role of the ventral basal ganglia in sleep–wake regulation associated with motivation (Supplementary Fig. 10).

Recently, numerous studies have discovered a crucial role of the mesolimbic dopaminergic system in sleep–wake regulation [22, 23, 29]. VTA dopaminergic neurons and glutamatergic neurons promote arousal [22, 23], while GABAergic VTA neurons decrease arousal [34]. Furthermore, VP-to-VTA projections regulate several motivation-related behaviors [21]. In the current study, we report that VP GABAergic neurons functionally connect to VTA dopaminergic, glutamatergic, and GABAergic neurons. In vivo stimulation of VP GABAergic neurons increased VTA dopaminergic neuronal activities, and stimulation of VP^GABA^-VTA projections induced arousal. Further analysis of this circuit mechanism showed that VP GABAergic neurons predominantly inhibited VTA GABAergic neurons, the latter of which inhibit wake-promoting dopaminergic and glutamatergic neurons. Although VP GABAergic neurons formed some direct inhibitory connections with VTA wake-promoting dopaminergic and glutamatergic neurons and decreased the firing of these neurons, the general effect in vivo was a disinhibition of these neurons and induction of arousal. Our previous study demonstrated that NAc D_1_R neurons promote arousal through disinhibition of VTA dopaminergic neurons, but that NAc D_1_R neurons exerted sparse inhibitory connections onto VTA dopaminergic neurons [25]. Moreover, in the present study, pretreatment with dopaminergic antagonists completely blocked the wakefulness induced by the activation of VP GABAergic neurons, further demonstrating that activation of VTA dopaminergic neurons plays a crucial role in mediating arousal via VP GABAergic neurons.

In the present study, we also found that VP GABAergic neurons regulated arousal through the LH pathway. Several subtypes of neurons in the LH regulate sleep–wake behaviors [27]; LH orexinergic neurons are necessary for maintaining arousal, while several subtypes of GABAergic neurons promote sleep [35]. In our present study, in vivo stimulation of VP GABAergic neurons increased the activity of LH orexinergic neurons but not MCH neurons. We speculate that activation of VP GABAergic neurons may inhibit LH inhibitory interneurons and disinhibit wake-promoting neurons (i.e., orexinergic neurons) [36], which would explain why we found increased c-Fos expression in LH orexinergic neurons following activation of VP GABAergic neurons. Indeed, a subset of LH GABAergic neurons send inputs to the sleep-promoting ventrolateral preoptic area and regulate wakefulness [37], whereas another subset sends inputs to other wake-promoting regions, including the tuberomammillary nucleus, ventral periaqueductal gray, and locus coeruleus, all of which may promote sleep. Nevertheless, certain subtypes of inhibitory interneurons may also receive VP GABAergic projections and regulate arousal. Collectively, our findings suggest that the VP^GABA^-LH circuit involved in controlling arousal may be partially mediated through activating LH wake-promoting orexinergic neurons.

Interestingly, a previous study has shown that chemogenetic activation of basal forebrain (BF) GABAergic neurons also promotes arousal [38]. Although the BF and VP are located nearby to one another, they have distinct projections and functions. By synthesizing our present findings with those of previous studies, we suspect that GABAergic neurons in both the VP and BF are important in regulating arousal, which may each contribute to different higher-order brain functions. In our present study, we also found that the population activity of VP GABAergic neurons decreased following prolonged wakefulness but was still higher than that during NREM sleep, suggesting that VP GABAergic neurons may play a role in maintaining wakefulness. Interestingly, the calcium activity of VP GABAergic neurons was also increased during NREM-to-REM transitions, but chemogenetic or optogenetic activation of these neurons only promoted arousal, which is consistent with previous findings on the function of NAc D_1_R neurons [25]. We suspect that several neurons in wake-promoting nuclei are activated during both wakefulness and REM sleep, but not NREM sleep.

The major input to the VP originates from the NAc. Our previous data have shown that NAc D_1_R neurons regulate wakefulness, whereas NAc D_2_R/A_2A_R neurons projecting to the VP regulate sleep [12, 25]. However, although both NAc D_1_R and D_2_R neurons are GABAergic neurons, they exhibit opposing functions. Due to less than 3% colocalization of D_1_R- and D_2_R-expressing fibers in the VP [39], we hypothesize that NAc D_1_R and D_2_R neurons each project to distinct targets in the VP. Indeed, a recent study showed that VP GABAergic neurons receive equal inputs from NAc D_1_R and D_2_R neurons [26]. However, NAc D_2_R neurons may specifically target VP wake-promoting neurons (e.g., wake-promoting GABAergic projection neurons), whereas NAc D_1_R neurons may specifically target VP GABAergic interneurons. Our present results have revealed that VP GABAergic neurons are wake-promoting, but there are still some GABAergic interneurons in the VP that may receive NAc D_1_R neurons inputs, which may decrease arousal. Further studies should clarify how NAc D_1_R/D_2_R neurons target specific VP neuronal subtypes and elucidate the differential roles of VP glutamatergic and cholinergic neurons in sleep–wake regulation.

The VP plays an active role in integrating information in the ventral basal ganglia, which is a key component in the brain for generating motivation-related behaviors, including locomotion, reward, learning/memory, addiction, and aversion. Inhibition of NAc D_2_R axonal terminals to the VP is sufficient to enhance motivation [40], which is consistent with another previous finding that inhibition of NAc D_2_R terminals in the VP promotes behavioral arousal [12]. An optogenetic study showed that activity in VP GABAergic neurons projecting to the VTA drives positive reinforcement [21], indicating that VP-VTA GABAergic projection neurons promote motivation-related behavior via influencing VTA dopaminergic neurons. Our present results support these findings that arousal induced by VP GABAergic neurons projecting to the VTA contributed to motivation-related behavior. In contrast, the VP also sends inputs to the LH, which plays a key role in feeding behaviors [41], but activation of VP GABAergic neurons in our present study did not significantly influence food intake, indicating that non-feeding-related subtypes of LH neurons may have been preferentially activated. Taken together, previous studies and our present findings clearly elucidate a NAc-VP-VTA/LH circuit in modulating arousal, which also significantly influences motivation-related behaviors; hence, this elucidated circuit constitutes a crucial link between arousal and motivation.

VP-mediated encoding of and contributions to response vigor are specific to the ability of incentive cues to invigorate reward-seeking behaviors, upon which reward delivery is contingent [42]. A recent study showed that stimulating VP GABAergic neurons facilitated cocaine-seeking and that cued seeking increased the number of calcium-signaling events in VP GABAergic neurons [35]. These findings strongly support our present conclusion that increasing the activity of VP GABAergic neurons is necessary for controlling and maintaining wakefulness, which may provide a foundation for motivation-related and drug-seeking behaviors. Several VP-implicated neuropsychiatric disorders induce sleep–wake alterations, indicating that the NAc-VP-VTA/LH circuit may represent a potential therapeutic target for sleep disorders, especially those related to neuropsychiatric disorders. For example, reward-specific firing in the VP is present in a greater proportion and arises soon following reward delivery [43], which may explain aberrant arousal in reward situations. As the VP regulates motivation and cognition, adaptations in this system may contribute to the mood and mnemonic disruptions that can accompany Parkinson’s disease [44].

In conclusion, we found a key role of VP neurons in integrating information from the ventral basal ganglia and in promoting arousal and motivation-related behaviors through a ventral midbrain pathway.

## Materials and methods

### Animals

Vgat (Slc32a1 or Viaat)-Cre mice (10–14 weeks old), weighing 22–26 g were obtained from the Jackson Laboratory (Stock No: 017535). GAD67-GFP knock-in mice [45] were obtained from Yuchio Yanagawa. Only male mice were used for experiments. Mice were group-housed (3–5 per cage) under a 12-h (07:00–19:00) light–dark cycle within a colony room at 22 °C (humidity ≈ 60%; illumination intensity ≈ 100 lux). Food and water were provided ad libitum. All experimental protocols were approved by the Fudan University Animal Care and Use Committee.

### Chemicals and drug administrations

CNO (C4759, LKT, USA) at 1 mg/kg was intraperitoneally (i.p.) injected at 09:00 or 21:00. SCH23390 (Sigma, USA) at 0.03 mg/kg and raclopride (Sigma, USA) at 2 mg/kg were freshly prepared and i.p. administered at 15 min before CNO administration. All of the drugs were injected at a volume of 10 ml/kg of body weight.

### EEG/EMG electrode-implantation surgery

Briefly, mice were anesthetized under 1.5% isoflurane in oxygen at a 0.8-LPM flow rate. Two stainless-steel screws, which served as EEG electrodes, were implanted into the skull above the right cortex (coordinates: anteroposterior [AP]: ±1.0 mm, mediolateral [ML] + 1.5 mm), and two EMG electrodes were implanted in the dorsal neck musculature to monitor muscular activity, as previously described [46, 47]. All EEG electrodes were fixed to the skull via dental cement.

### Viral injections and fiber implantations

AAV_10_-hSyn-DIO-hM3Dq/hM4Di-mCherry, AAV_10_-hSyn-DIO-ChR2-mCherry, and AAV_2_-hSyn-DIO-GCaMP6f constructs (1–2 × 10^12^ genomic particles/mL) were used in chemogenetic, optogenetic, and fiber-photometry experiments, respectively [6, 25]. Mice were anesthetized with sodium pentobarbital and placed in a stereotaxic apparatus (RWD, Shenzhen, China), followed by microinjection of 100–200 nL of construct (AAV_10_-DIO-hM3Dq/hM4Di-mCherry or AAV_10_-DIO-mCherry) into the bilateral VP (AP: + 0.14 mm, ML: ± 1.5 mm, DV: −4.9 mm) or 200 nL of AAV_2_-hSyn-DIO-GCaMP6f into the left VP. After injections, mice used for in vivo optogenetic stimulation experiments were bilaterally implanted with optical fibers (fiber core, 200 μm; 0.37 numerical aperture [NA], Newdoon, Hangzhou, China) above the VP (AP: +0.14 mm, ML: ±1.5 mm, DV: −4.6 mm), LH (AP: −0.94 mm, ML: ±1.1 mm, DV: −4.0 mm), VTA (AP:−3.64 mm, ML: ±0.5 mm, DV: −3.8 mm), LHb (AP: −1.58 mm, ML: ±0.5 mm, DV: −2.2 mm), or MD (AP: −2.05 mm, ML: ±0.5 mm, DV: −3.2 mm). For fiber photometry, a unilateral fiber was implanted into the left VP (AP: +0.14 mm, ML :±1.5 mm, DV: −4.8 mm). Mice were housed for at least 2 weeks after injections for complete recovery. After behavioral tests, mice were sacrificed to verify viral expression.

### EEG recordings and analysis

EEG recordings and analysis were performed as previously described [48–50]. Cortical EEG and neck EMG signals were amplified and filtered (Biotex, Kyoto, Japan. EEG, 0.5–30 Hz; EMG, 20–200 Hz). Each frequency band in the EEG was calculated via SleepSign software using the fast Fourier transformation (FFT) method. For FFT analysis, Fourier transformation was used to calculate the power variable (*uV*^2^), and absolute power spectra of the EEG data were computed every 4 s over a 0–25-Hz window with 0.25-Hz resolution. Absolute power spectra were transferred into relative changes by taking a synchronization value as 100%. For sleep-stage analysis, EEGs and EMGs were automatically classified using 4-s epochs for wakefulness, REM sleep, and NREM sleep according to standard criteria.

### Fiber photometry

Fiber-photometry experiments were performed in both light and dark periods as previously described [24, 25]. Photometry data were exported to MATLAB Mat files from Spike2 for further analysis. We derived the value of the photometry signal (*ΔF*/*F*) by calculating (*F*–*F*_0_)/*F*_0_, where *F*_0_ is the median fluorescent signal. The averaged *ΔF*/*F* was calculated during all times of sleep–wake states. For analyzing state transitions, we determined each state transition and aligned *ΔF*/*F* in a ± 50-s window around each point that was calculated. The average peak of the *ΔF*/*F* was selected and compared for different sleep stages.

### Optogenetic stimulation during polygraphic recordings

For in vivo light stimulation, light-pulse trains were generated via a laser stimulator (SEN-7103, Nihon Kohden, Japan) and output through an isolator (ss-102J, Nihon Kohden, Japan). A rotating optical joint (FRJ_FC-FC, Doric Lenses, Canada) was used to relieve torque and was attached to the external end of the optical fiber. For acute photostimulation, each stimulation epoch was applied 20 s after identifying a stable NREM or REM sleep event by real-time online EEG/EMG analysis. Light-pulse trains (5-ms duration each) were programmed and conducted during the light period, when mice are inactive. The cut-off line for stage transitions was 60 s after the laser was turned on. For chronic photostimulation, programmed light-pulse trains (5-ms pulses at 20 Hz, with 10-s on/ 20-s off for 120 cycles) were used from 09:00 to 10:00. EEG/EMG recordings during the same period on the previous day served as a baseline control. Power intensities of blue or yellow light at the tip of the optical fiber were calibrated to emit 3–7 mW [51].

### Behavioral tests

#### Elevated plus-maze test (EPM)

Each mouse was placed in the center of the intersecting arms, facing an open arm, and was allowed to explore the apparatus for 5 min. The time spent in each arm was recorded by an experimenter who was blinded to the treatment [52]. Anxiety-like behavior was determined by measuring the time spent in the open arms.

#### Open-field test (OFT)

Mice were gently placed in the center of the field, and movement was recorded for 5 min with a video-tracking system. The time spent in the center of the arena (defined as a 20 × 20 cm zone in the center of the apparatus) was measured [52].

#### Light-dark box test

Each mouse was released in the center of the light compartment and was allowed to explore the arena for 5 min. The number of entries and time spent in the white compartment were recorded [53].

#### Tail-suspension test

Mice were suspended by the tail from a metal rod using adhesive tape. The rod was fixed 45 cm above the ground. The test session was recorded for 5 min, and the immobility time was determined by an observer who was blinded to the treatment conditions [52].

#### Feeding-behavior assays

Mice were individually housed in home cages with a mild food restriction by removing chow food during the final 8 h before lights were turned off (19:00). All of the tests were performed using a counter-balanced within-subjects design. Mice received i.p. injections of either vehicle or CNO (1 mg/kg) at 19:00, 15 min before access to ~5 g of fresh standard chow. Food intake was measured at 3 and 12 h after CNO injection [54].

### Immunohistochemistry

Immunohistochemistry was performed as described previously [25, 52, 55]. Mice were deeply anesthetized at 1.5 h after CNO administration or 0.5 h after light stimulation and were then perfused intracardially with phosphate-buffered saline (PBS) followed by 4% paraformaldehyde (PFA). After each brain sank in a 30% sucrose solution, coronal slices (30 μm) were cut into four series via a microtome (CM1950, Leica, Germany).

For c-Fos staining, incubation with a primary antibody was performed at 4 °C for 48 h (rabbit anti-c-Fos, 1:10,000; ABE457, Millipore, USA). Sections were then washed in PBS and incubated with a biotinylated secondary antibody (donkey anti-rabbit, 1:1000, Vector Labs, USA) for 2 h at room temperature, followed by treatment with an avidin-biotin-peroxidase complex (Vector Labs, USA) for 1 h. Finally, the sections were immersed in a DAB and nickel-ammonium-sulfate solution (Vector Labs, USA) for staining. For mCherry/TH/orexin/MCH staining, the following primary antibodies were used: rabbit anti-DsRed (1:10,000; Clontech, Cat# 632496), mouse anti-TH (1:1000, T2928, Sigma, USA), goat anti-orexin-A (1:500, sc-8070, Santa Cruz Biotechnology, USA), and goat anti-MCH (1:800, sc14507, Santa Cruz Biotechnology, USA).

### In vitro electrophysiology

In vitro electrophysiological experiments were performed 3–4 weeks after AAV-ChR2 injections in Vgat-Cre mice or Vgat-Cre:GAD67-GFP double-transgenic mice. Mice were anesthetized and perfused transcardially with ice-cold modified aCSF saturated with 95% O_2_ and 5% CO_2_ and that contained the following (in mM): 215 sucrose, 26 NaHCO_3_, 10 glucose, 3 MgSO_4_, 2.5 KCl, 1.25 NaH_2_PO_4_, 0.6 Na-pyruvate, 0.4 ascorbic acid, and 0.1 CaCl_2_. Brains were then rapidly removed, and acute coronal slices (300 μm) containing the VP, LH, or midbrain were cut on a vibratome (VT1200, Leica, Germany) in ice-cold modified aCSF. Next, slices were transferred to a holding chamber containing normal recording aCSF (in mM): 125 NaCl, 26 NaHCO_3_, 25 glucose, 2.5 KCl, 2 CaCl_2_, 1.25 NaH_2_PO_4,_ and 1.0 MgSO_4_. In Vgat-Cre::GAD67-GFP mice, GAD67-GFP neurons were identified based on their GFP expression. Recordings were performed in regions with bright mCherry fluorescence. Recording pipettes were filled with an internal solution containing the following (in mM): 105 potassium gluconate, 30 KCl, 10 phosphocreatine, 4 ATP-Mg, 0.3 EGTA, 0.3 GTP-Na, and 10 HEPES (pH 7.3, 285–300 mOsm). In some experiments, 0.1% biocytin (vol/vol, Sigma, USA) was included in the internal solution. Recordings were conducted in the whole-cell or cell-attached configuration using a Multiclamp 700B amplifier (Axon Instruments, USA). Signals were filtered at 4 kHz and digitized at 10 kHz with a DigiData 1440 A (Axon Instruments, USA). Data were acquired and analyzed with pClamp10.3 software (Axon Instruments, USA).

We identified VTA dopaminergic neurons (TH+) using the following three criteria: (1) resting membrane potential more depolarized than −70 mV and fired spontaneous action potentials at frequencies lower than 10 Hz or were quiescent; (2) a pronounced hyperpolarization-activated inward current (*I*_h_); and (3) broad action potentials (spike duration >1.3 ms) with a pronounced after-hyperpolarization (Supplementary Fig. 5f). Neurons that did not satisfy these criteria were identified as TH− neurons (Supplementary Fig. 5j), as previously described [12, 25, 56, 57].

Responses were evoked by 5-ms light flashes (473 nm, 1–100 Hz) delivered from a microscope-mounted blue LED (Lumen Dynamics, Canada) through the objective lens directed onto the slice. The power of the LED light was 3–5 mW. In the voltage-clamp mode, cells were held at −70 mV. When needed, 25 μM of d- (−)-2-amino-5-phosphonopentanoic acid (d-APV), 5 μM of 6-cyano-7- nitroquinoxaline-2,3-dione (CNQX), and 10 μM of SR (gabazine) and CGP55845 were added to block NMDA, AMPA, GABA_A_, and GABA_B_ receptors, respectively. Cells with R_a_ changes over 20% were discarded.

### Single-cell RT-PCR

At the end of each electrophysiological recording, single-cell PCR from the recorded cell was performed as described previously [25, 58]. The presence of mRNAs coding for TH or shVglut2 was determined for the recorded cells. TH was amplified in the first round with the TH up (5′-GCT GTC ACG TCC CCA AGG TT-3′) and TH lo (5′-AAG CGC ACA AAG TAC TCC AGG-3′) primers. In the second amplification round, we used TH up2 (5′-CGC GGA ACC TGG GAA CCC-3′) and TH lo2 (5′-TCC TGC CAG TGG CCT CTG-3′) primers. Primers were used to generate a PCR product of 190 base pairs (b.p.) in size. The Vglut2 primers were multiplex forward (5′-TGTTCTGGCTTCTGGTGTCTTACGAGAG-3′), and reverse (5′-TTCCCGACAGCGTGCCAACA-3′) primers, as well as nested foward (5′-TCAACAACAGCACCATCCAC-3′), and nested reverse (5′-GGGCTCTCGTAAGACACCAG-3′) primers. Primers were used to generate a PCR product of 315 b.p. in size.

### Statistical analysis

Data are expressed as the mean ± standard error of the mean (SEM). Statistical significance was assessed using two-tailed paired Student’s *t* tests to compare the total sleep amounts and behavioral results between the two groups. One-way, two-way, or repeated-measures analyses of variance (ANOVAs) were used to compare sleep amounts, followed by pairwise comparisons via Tukey post-hoc tests. EEG power spectra were compared using one-way ANOVAs followed by Fisher’s PLSD tests or via non-paired, two-tailed Student’s *t* tests. Two sets of frequencies were analyzed by chi-square tests. A two-tailed *P* value <0.05 was considered to be statistically significant. All of the data were analyzed using Prism 8.0 software.

## Supplementary information

Supplementary figures and legends

## Data Availability

The data that support the findings of this study are available from the corresponding author upon request.

## Code availability

Data analyses were conducted in Matlab using scripts available from the corresponding author upon reasonable request.
