# Supplementary material for: Ventral pallidal GABAergic neurons control wakefulness associated with motivation through the ventral tegmental pathway
Source: Mol Psychiatry. 2020 Oct 14;26(7):2912–28. doi: 10.1038/s41380-020-00906-0 (PMC8505244; doi:10.1038/s41380-020-00906-0)
Supplement: Supplementary file 1 — Supplementary figures and legends [file 41380_2020_906_MOESM1_ESM.docx]

**Supplementary figures and legends**

**Ventral pallidal GABAergic neurons control wakefulness associated with motivation through the ventral tegmental pathway**

Ya-Dong Li^1*^, Yan-Jia Luo^1*^, Wei Xu^1^, Jing Ge^1^, Yoan Cherasse^2^, Yi-Qun Wang^1^, Michael Lazarus^2^, Wei-Min Qu^1^ & Zhi-Li Huang^1^

^1^Department of Pharmacology, School of Basic Medical Sciences; State Key Laboratory of Medical Neurobiology and MOE Frontiers Center for Brain Science, and Institutes of Brain Science, Fudan University, Shanghai 200032, China

^2^International Institute for Integrative Sleep Medicine (WPI-IIIS), University of Tsukuba, 1-1-1 Tennodai, Tsukuba, Ibaraki 305-8575, Japan.

*These authors contributed equally to this work

Correspondence and requests for materials should be addressed to Zhi-Li Huang ([huangzl@fudan.edu.cn](mailto:huangzl@fudan.edu.cn)) or Wei-Min Qu ([quweimin@fudan.edu.cn](mailto:quweimin@fudan.edu.cn)).


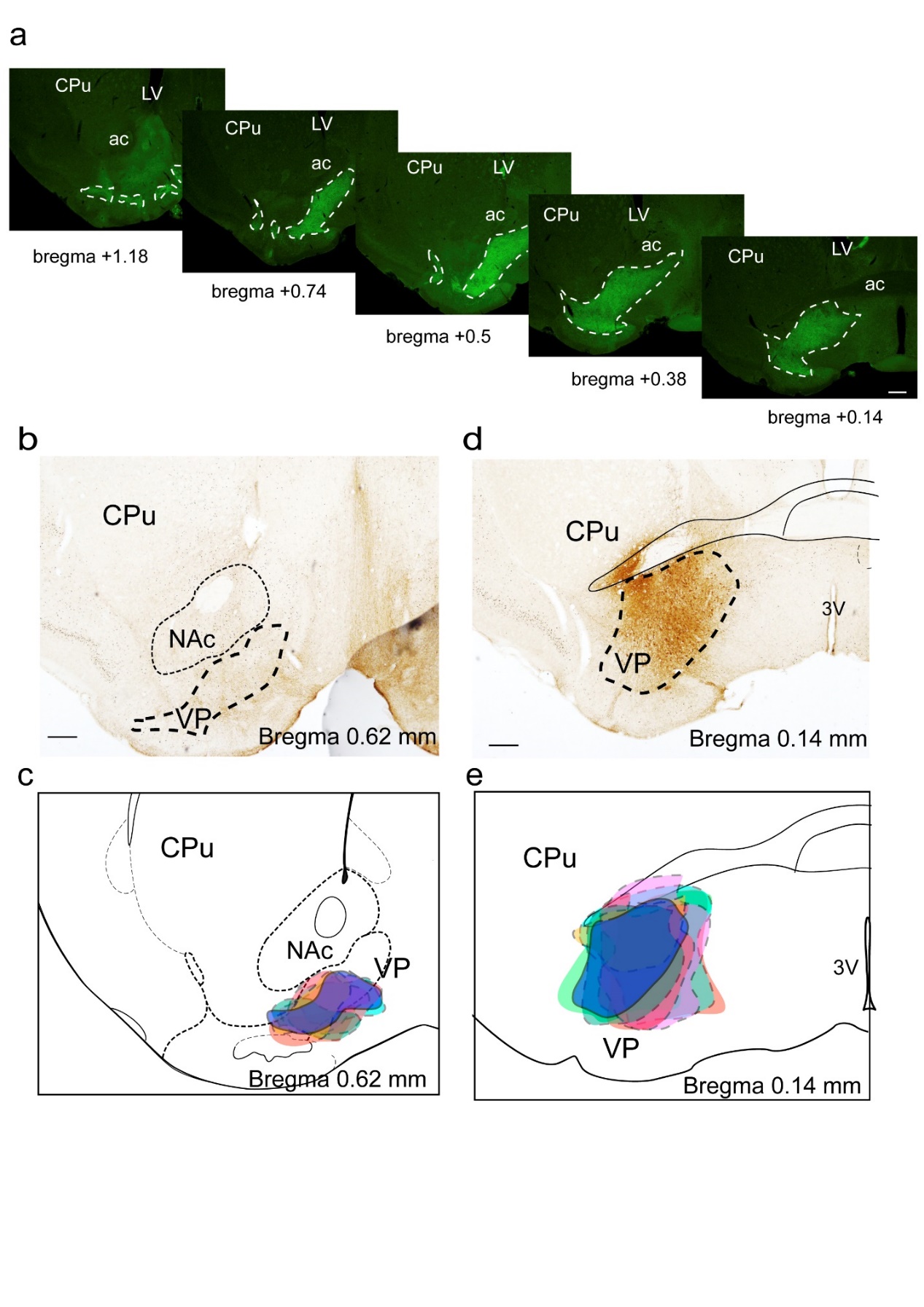


**SI Fig. 1** **Substance P showing the location of the VP and viral expression in chemogenetically activated mice.** (a) Immunochemical staining of substance P, showing the boundary of the VP. (b, d) Typical example of DIO-hM3Dq-mCherry expression in the VP (Bregma = 0.62 in b and bregma = 0.14 in d; Scale bar = 200 μm). (c, e) Coronal brain-atlas diagram showing the approximate distribution of nine hM3Dq-mCherry-infected regions, where locations for each individual mouse are indicated by different colored outlines (Bregma = 0.62 in b and bregma = 0.14 in d).


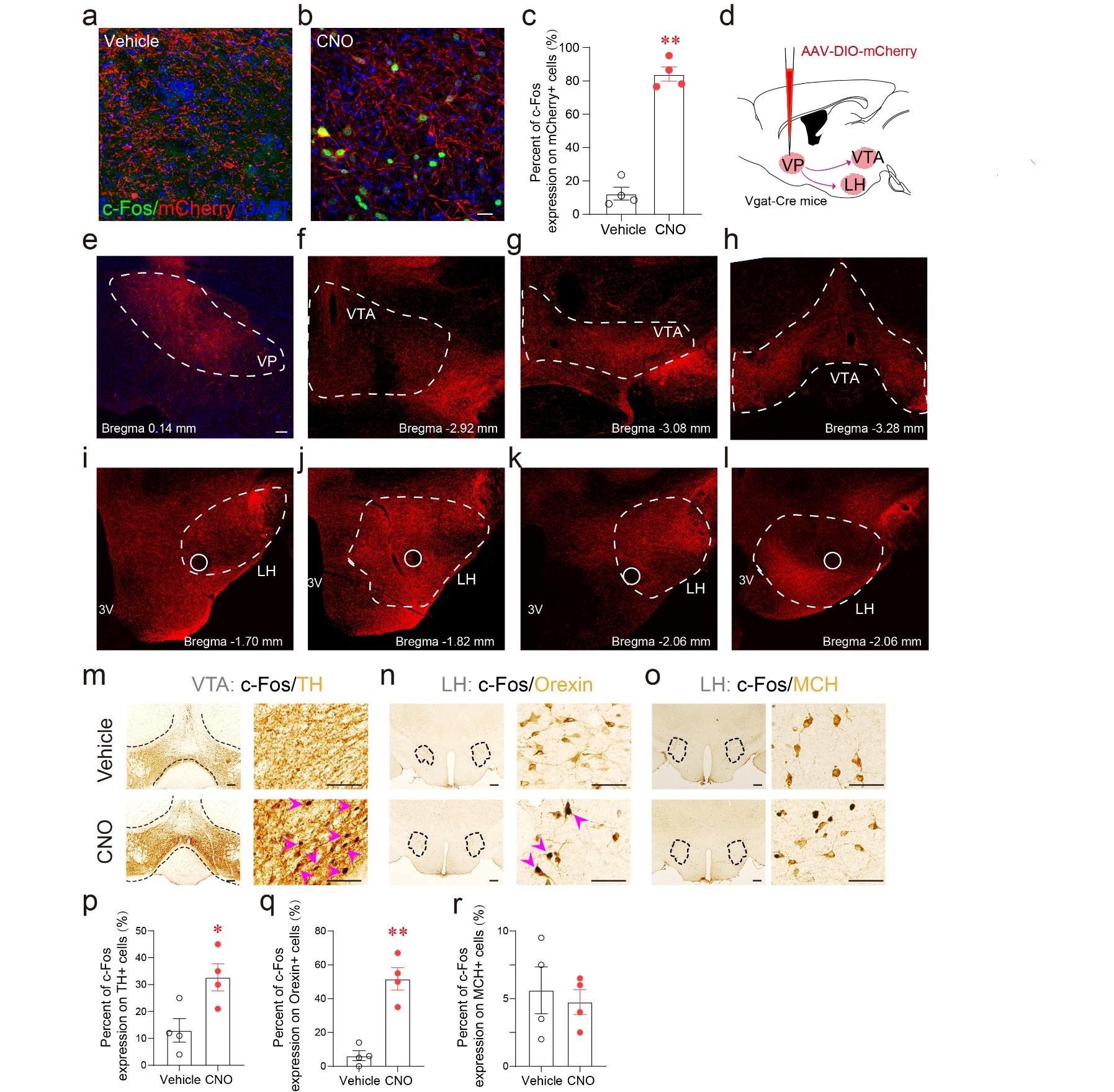


**SI Fig. 2** **VP GABAergic neurons innervated VTA^TH^ neurons and LH°^rexin^ neurons.** (a-b) C-Fos expression in the VP of hM3Dq-mCherry mice after vehicle (a) or CNO (b) treatment, Scale bar = 20 μm. (c) CNO administration increased c-Fos expression on mCherry+ cells by 6.8-fold. ***P* < 0.01 by unpaired *t*-test, n = 4 mice. (d) Diagram of anterograde tracing. AAV-DIO-mCherry was injected into the VP in Vgat-Cre mice. (e) AAV-DIO-mCherry infected cells were located in the VP. (f–h) mCherry terminals of VP GABAergic neurons in the VTA. (i–l) mCherry terminals of VP GABAergic neurons in the LH. Scale bar = 200 μm. (m–o) Double staining of c-Fos and TH (m) in the VTA, and orexin (n) or MCH (o) in the LH after CNO activation in hM3Dq Vgat-Cre mice. C-Fos/TH and c-Fos/Orexin double positive cells were indicated by pink arrows. Scale bar = 100 μm. (p–r) Quantification of c-Fos expression on TH+ cells (p), Orexin+ cells (p) and MCH+ cells, respectively. C-Fos expression in TH+ and Orexin+ neurons was increased by 1.7-fold and 8.8-fold, respectively. Data shown are the mean ± SEM (**P* < 0.05, ***P* < 0.01 by unpaired *t*-test, n = 4 mice).


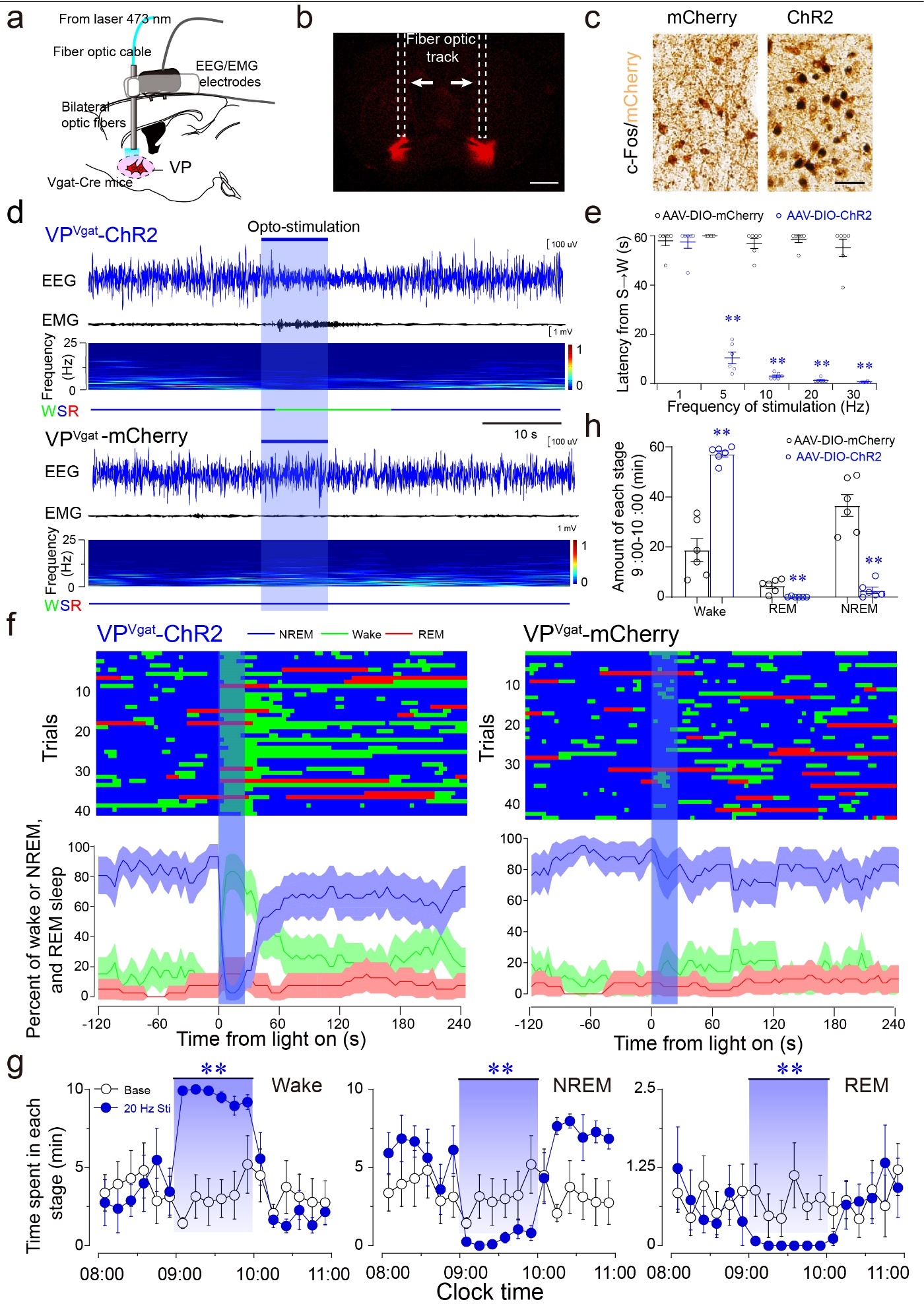


**SI Fig. 3 Optogenetic activation of VP GABAergic neurons induced and maintained wakefulness.** (a) Sagittal diagram for *in-vivo* optogenetic stimulation of VP GABAergic neurons in Vgat-Cre mice. (b) Fluorescence of mCherry expressed in the bilateral VP and tips of optical fibers within the VP. Scale bar = 1 mm. (c) Double-immunohistochemical staining with mCherry (light brown) and c-Fos (black) in the VP after light stimulation. Scale bar = 50 μm. (d) Optogenetic activation of VP GABAergic neurons induced an immediate transition from NREM sleep to wakefulness. Heatmap of EEG and representative traces of EEG and EMG recordings in a Vgat-Cre mouse expressing ChR2-mCherry (upper) or mCherry (below). Optogenetic stimulation immediately and significantly decreased EEG delta power and increased EMG activity, indicating a transition from NREM sleep to wakefulness. (e) The latency from NREM sleep to wakefulness decreased with increased stimulation frequency ranging from 5–30 Hz. (f) Sleep stage after blue-light stimulation. For each stimulation trial, EEG was analyzed for 6 min with a 2-minute baseline, and the light-on time was 20 s at 20 Hz. The percentages of NREM sleep, REM sleep, and wakefulness were analyzed during the short-stimulation experiment. (g) Time course of wake, REM sleep and NREM sleep before, during and after chronic optogenetic activation of VP GABAergic neurons. (h) Amount of wakefulness, NREM sleep, and REM sleep during 1-h optogenetic stimulation of VP GABAergic neurons (n = 6, ***P* < 0.01 using repeated-measures ANOVA, followed by Tukey *post hoc* test).


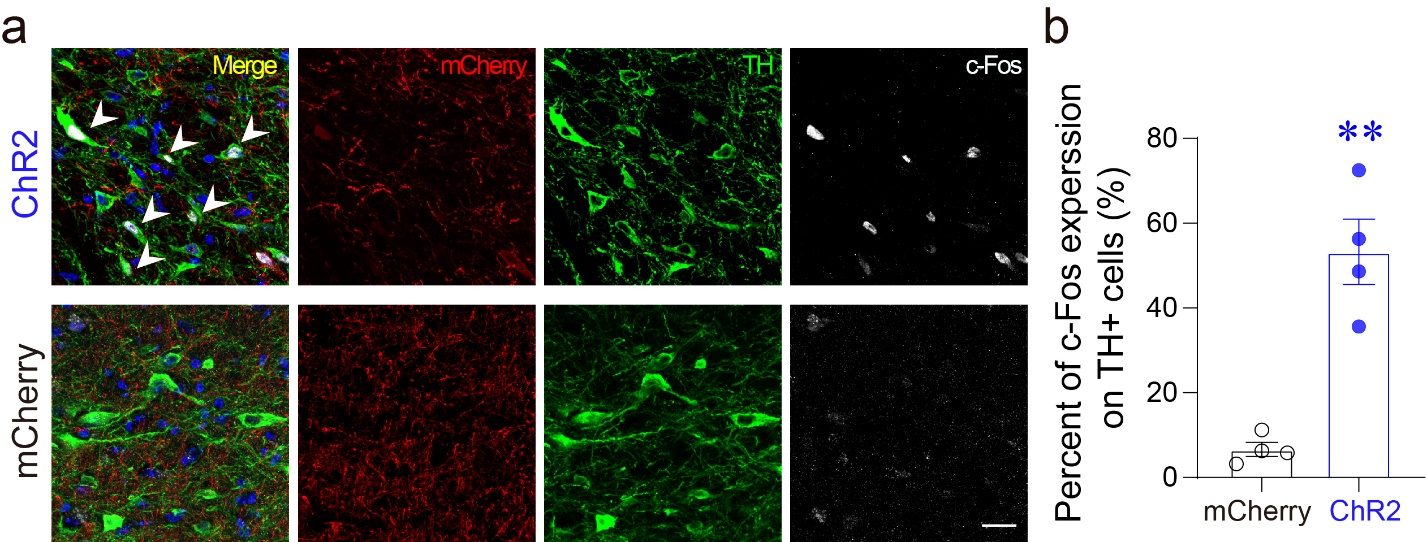


**SI Fig. 4 Optogenetic stimulation of VP^Vgat^-VTA projection increased c-Fos expression in TH+ neurons in the VTA.** (a) C-Fos expression in TH+ neurons after optogenetic stimulation of axonal terminals of VP GABAergic neurons in ChR2 or control mice. Scale bar = 20 μm (b) C-Fos expression in TH+ neurons was increased by nearly 10-fold after opto-stimulation. Data shown are the mean ± SEM (***P* < 0.01 by unpaired *t*-test, n = 4 mice).


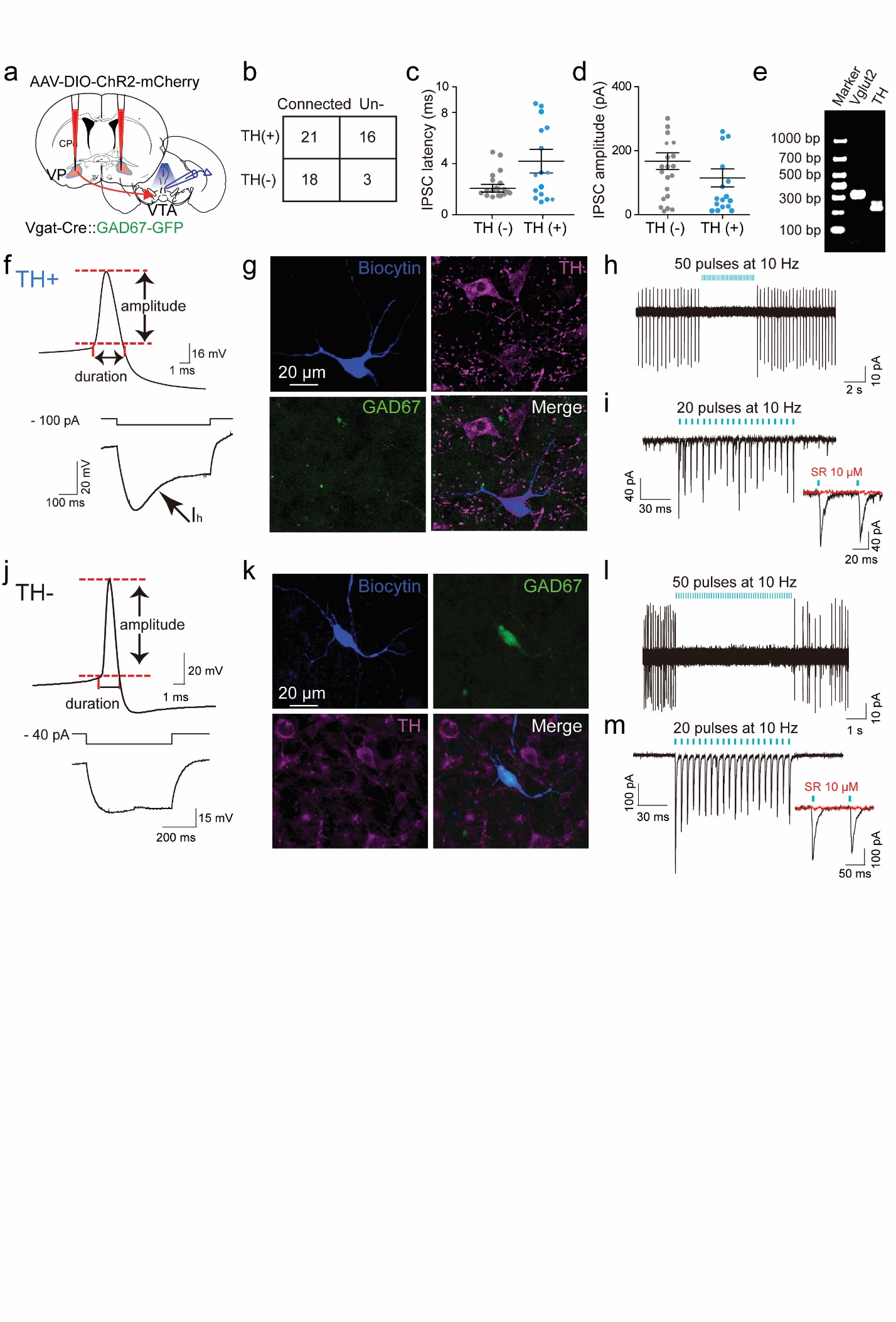


**SI Fig.** **5 VP GABAergic neurons innervated both TH-positive and TH-negative VTA neurons.** (a) Schematic showing injection of Cre-dependent ChR2 into the VP of a Vgat-Cre:GAD67-GFP double-transgenic mouse. Axonal terminals of the infected VP GABAergic neurons were optogenetically activated, and responses were recorded in VTA neurons. (b) Proportions of connected and unconnected TH-positive and TH-negative VTA neurons (n = 39 TH+ neurons, n = 21 TH- neurons, from 10 mice, Chi-square test; χ^2^ = 3.87, *P* = 0.049). (c) Latencies of light-evoked IPSCs in VTA TH-negative neurons and TH-positive neurons. (d) Amplitudes of light-evoked IPSCs in VTA TH-negative and TH-positive neurons. (e) Single-cell PCR showed that the GFP- neurons expressed Vglut2 or TH mRNA. (f–i) Typical examples of a connected biocytin-labeled neuron that was TH-positive-neuron responsive to light stimulation. The electrophysiological characteristics of a TH-positive neuron (f). Biocytin was overlapped with anti-TH, but not GFP (g). Blue-light pulses inhibited spontaneous spiking of a VTA TH-positive neuron. (h), and light-evoked IPSCs were blocked by SR (i). (j–m) Typical examples of a connected biocytin-labeled neuron that was TH-negative-neuron responsive to light stimulation. The electrophysiological characteristics of a TH-positive neuron (j). Biocytin was overlapped with GFP, but not anti-TH (k). Blue-light-train pulses inhibited spontaneous spiking of a VTA TH-negative neuron (l), and light-evoked IPSCs were blocked by SR (m).


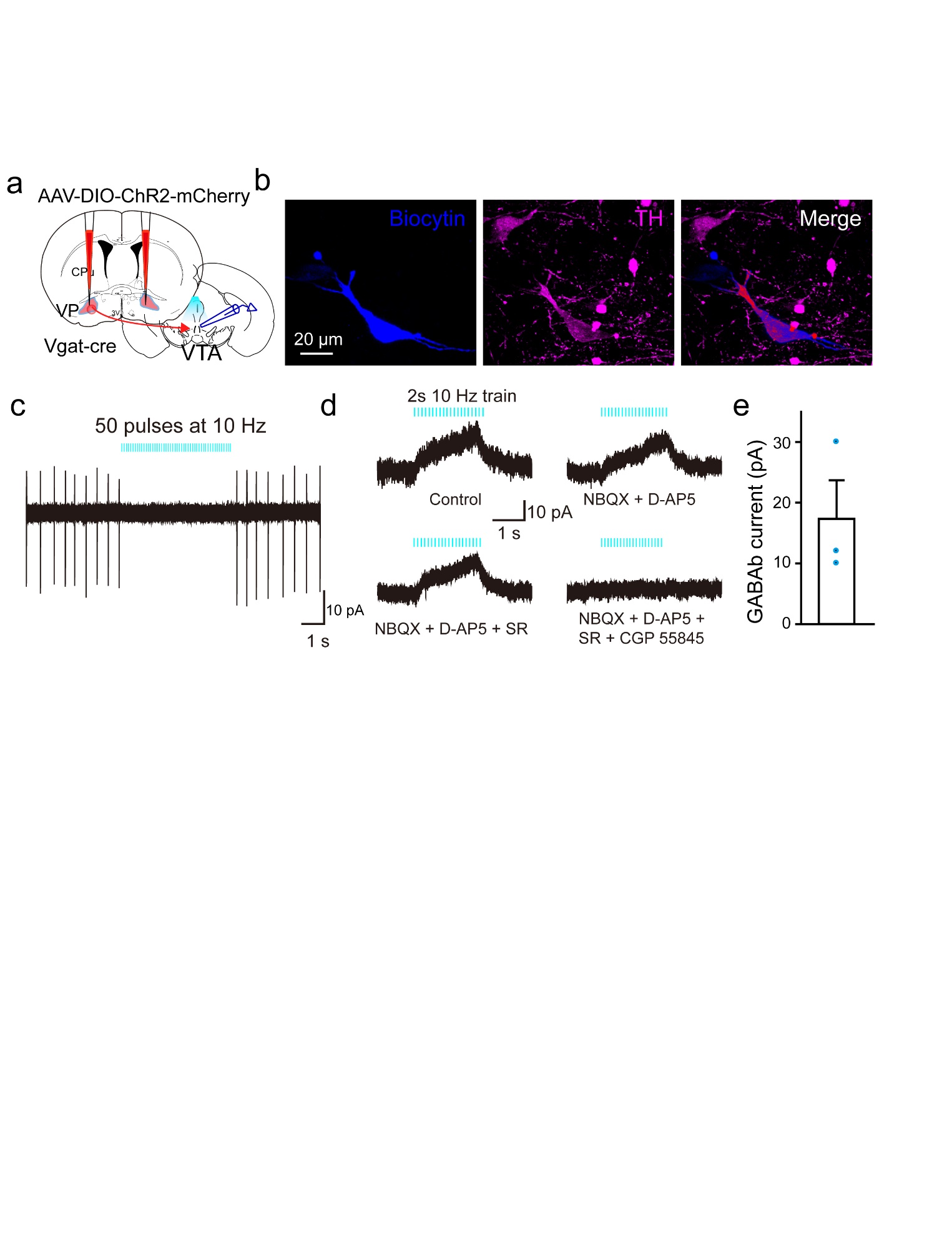


**SI Fig. 6 VP GABAergic neurons innervated a portion of VTA TH-positive neurons via GABA_B_ receptor signaling.** (a) Schematic showing injection of Cre-dependent ChR2 into the VP of a Vgat-Cre mouse. Terminals of the infected VP GABAergic neurons were optogenetically activated, and responses were recorded in VTA TH-positive neurons. (b) Biocytin was overlapped with anti-TH. (c) Blue-light-train pulses inhibited spontaneous spiking of a VTA TH+ neuron. (d) Light-evoked tonic currents were blocked by the GABA_B_R antagonist, CGP 58845, but not by NBQX, APV, or the GABA_A_R antagonist, SR. (e) Amplitudes of light-evoked tonic currents in VTA TH-positive neurons (n = 3 cells, scale bar = 20 μm).


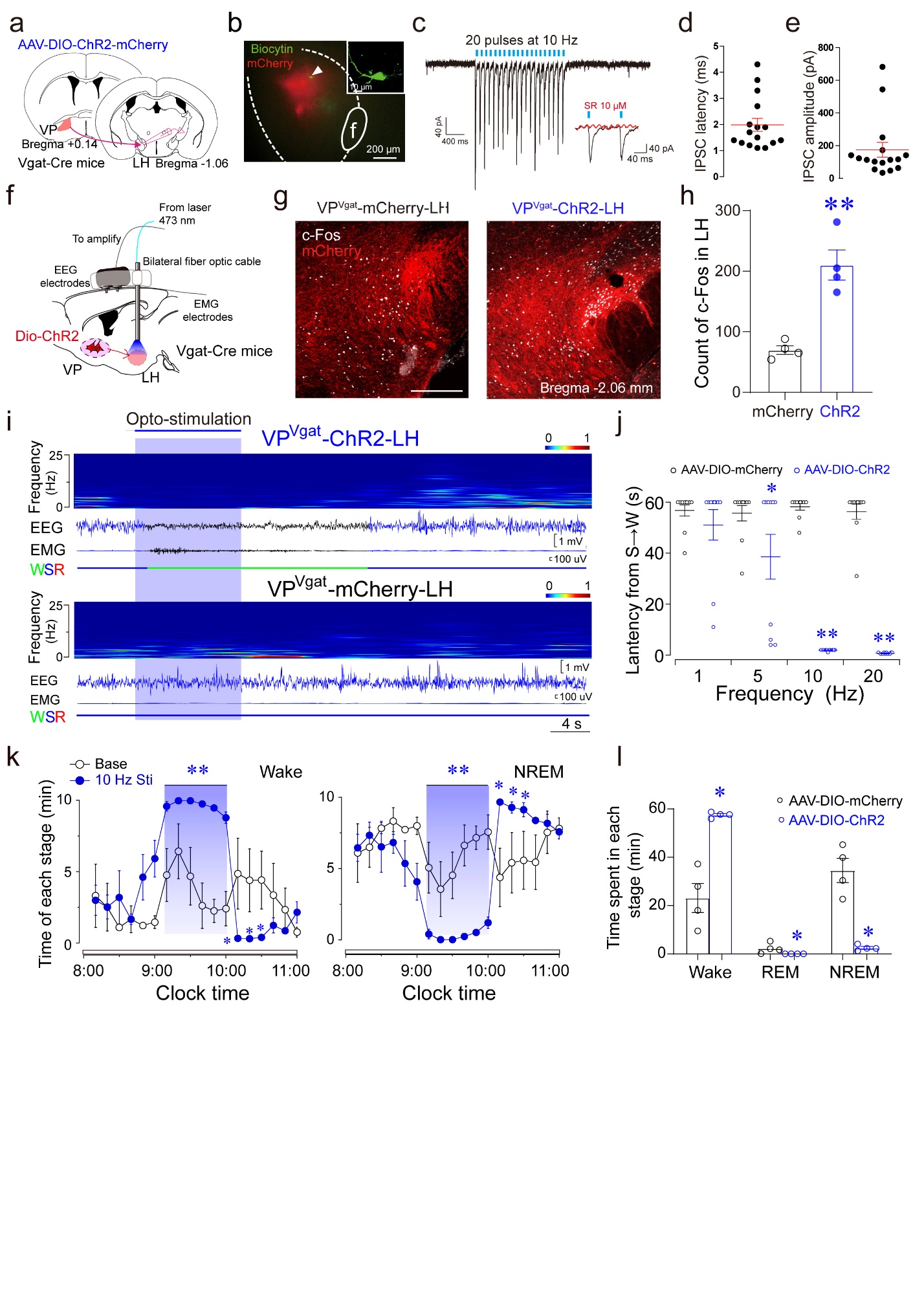


**SI Fig. 7 Activation of the VP^GABA^-LH pathway increased the amount of wakefulness.** (a) Schematic showing injection of Cre-dependent ChR2 into the VP of a Vgat-Cre mouse. Axonal terminals of the infected VP GABAergic neurons were optogenetically activated, and responses were recorded in LH neurons. (b) The mCherry terminals were found in the LH, and biocytin was located in the area of mCherry. (c) Light-evoked IPSCs were blocked by the GABA_A_R antagonist, SR. (d–e) Latencies and amplitudes of light-evoked IPSCs in LH neurons (n = 16 cells). (f) Sagittal diagram for *in-vivo* optical stimulation of the VP^GABA^-LH pathway. The AAV-DIO-hSyn-ChR2-mCherry construct was bilaterally expressed in the VP, and two optical fibers that were used for LH light delivery were implanted. (g) C-Fos expression after optogenetic stimulation of mCherry terminals in the LH. Scale bar = 200 μm. (h) C-Fos expression in the LH was increased by 2.1-fold after opto-stimulation. (n = 4, ** *P* < 0.01 using unpaired *t*-test) (i) EEG/EMG traces and EEG heatmap showing that optogenetic activation of the VP^GABA^-LH pathway induced wakefulness. Optogenetic stimulation of VP^GABA^-LH pathway induced decreasing delta power, and a low amplitude of EEG, with increasing EMG activity, in ChR2 mice, but not mCherry control mice. (j) Latency from NREM sleep to wakefulness decreased with increased stimulation frequencies. (k–l) One-hour photostimulation of the VP^GABA^-LH pathway increased the total duration of wakefulness but decreased NREM sleep and REM sleep during the inactive period (n = 4, * *P* < 0.05, ** *P* < 0.01 using repeated-measures ANOVA, followed by Tukey *post hoc* test).


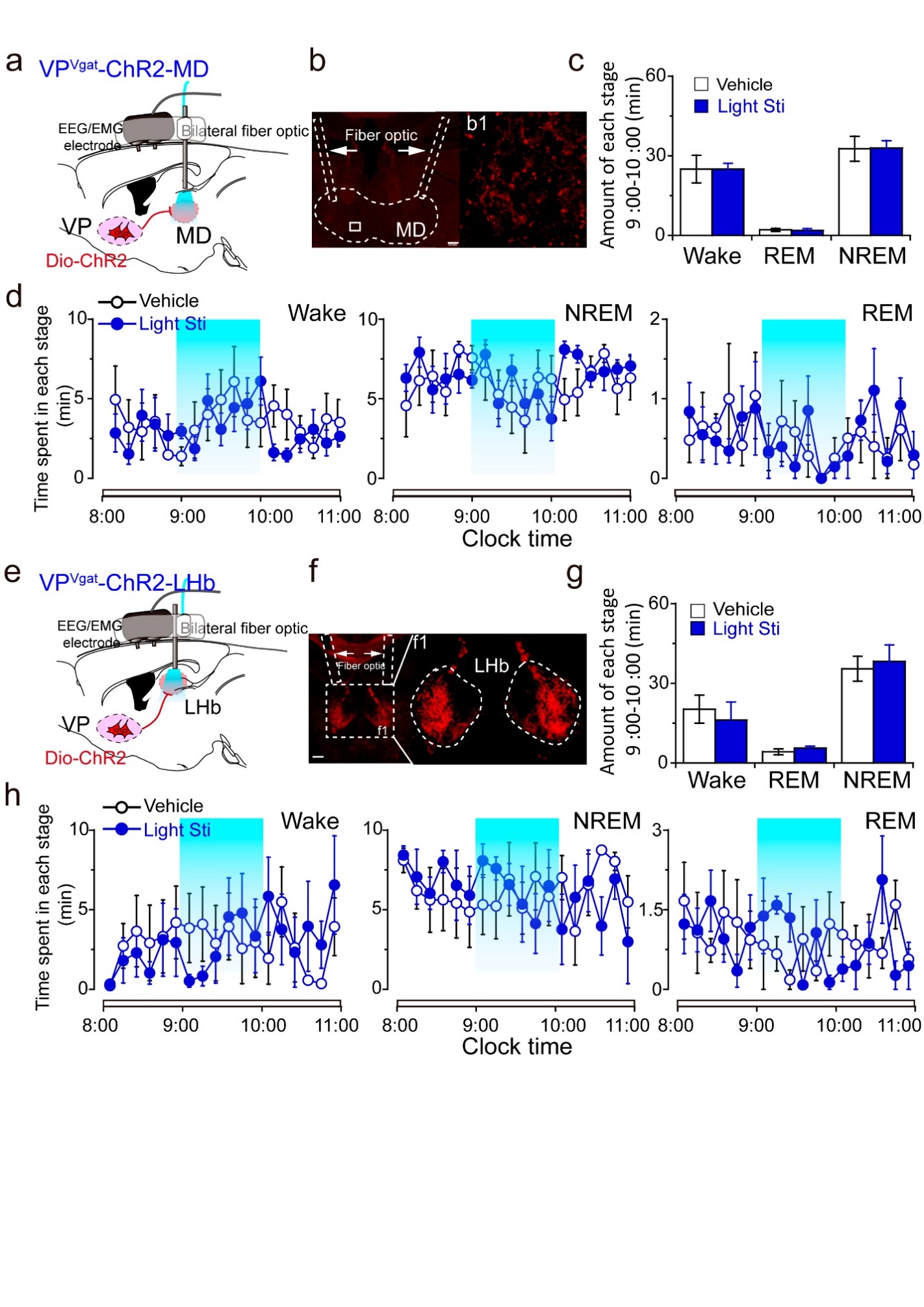


**SI Fig. 8 Optogenetic activation of the VP^GABA^-MD or VP^GABA^-LHb circuit did not alter sleep stages.** (a, e) Sagittal diagram of *in-vivo* optogenetic stimulation of the VP^GABA^-MD (a) or VP^GABA^-LHb pathway in Vgat-Cre mice (e). (b, f) Surgical implantation of bilateral optical fibers in the MD (b) and LHb (f). (c, g) Time spent in wakefulness, NREM sleep, and REM sleep during 1-h optogenetic photostimulation of the VP^GABA^-MD (c, n = 5) or VP^GABA^-LHb (g, n =4) pathway (*P* > 0.05 using repeated-measures ANOVA). (d, h) Time-course of optogenetic activation of the VP^GABA^-MD (d) or VP^GABA^-LHb (h) circuit (*P* > 0.05 using repeated-measures ANOVA. Scale bars = 200 μm).


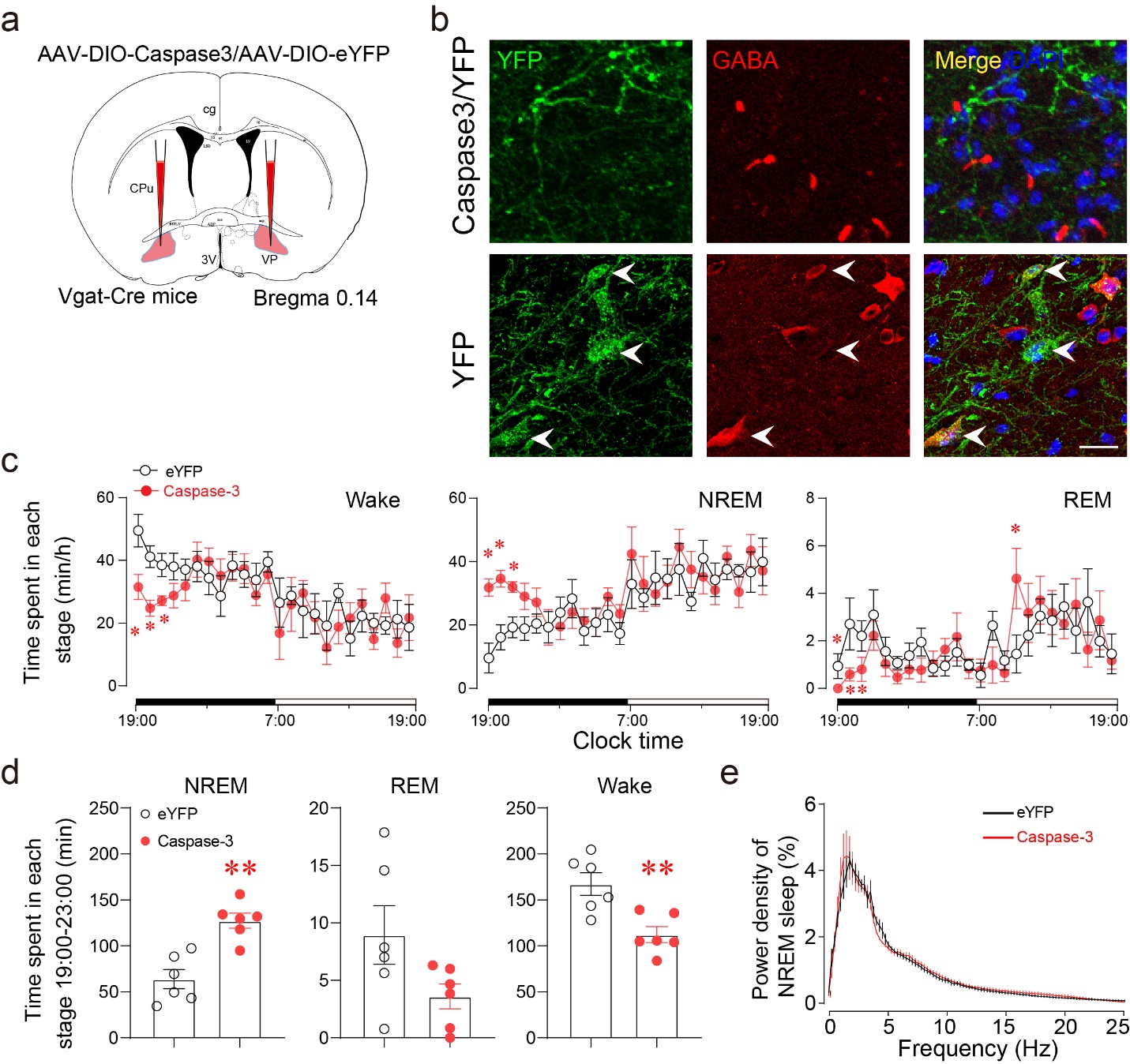


**SI Fig. 9 Lesioning of VP GABAergic neurons by Caspase-3 decreased wakefulness in the dark phase.** (a) Schematic diagram of Caspase-3 lesioning of VP GABAergic neurons. (b) GABA staining showed that VP GABAergic neurons were killed by Casapase-3. Scale bars = 20 µm. (c) Time course of wake, REM sleep and NREM sleep in mice with lesioning of VP GABAergic neurons by Caspase-3. (d) The cumulative time of wakefulness, NREM sleep, and REM sleep during 19:00–23:00 after GFP/Caspase-3 was expressed in the VP in Vgat-Cre mice. (e) EEG power of NREM sleep over 4 h in GFP/Caspase-3 mice. Data shown are the mean ± SEM (**P* < 0.05, ***P* < 0.01, two-way ANOVA followed by Tukey *post hoc* test, or unpaired *t*-test).


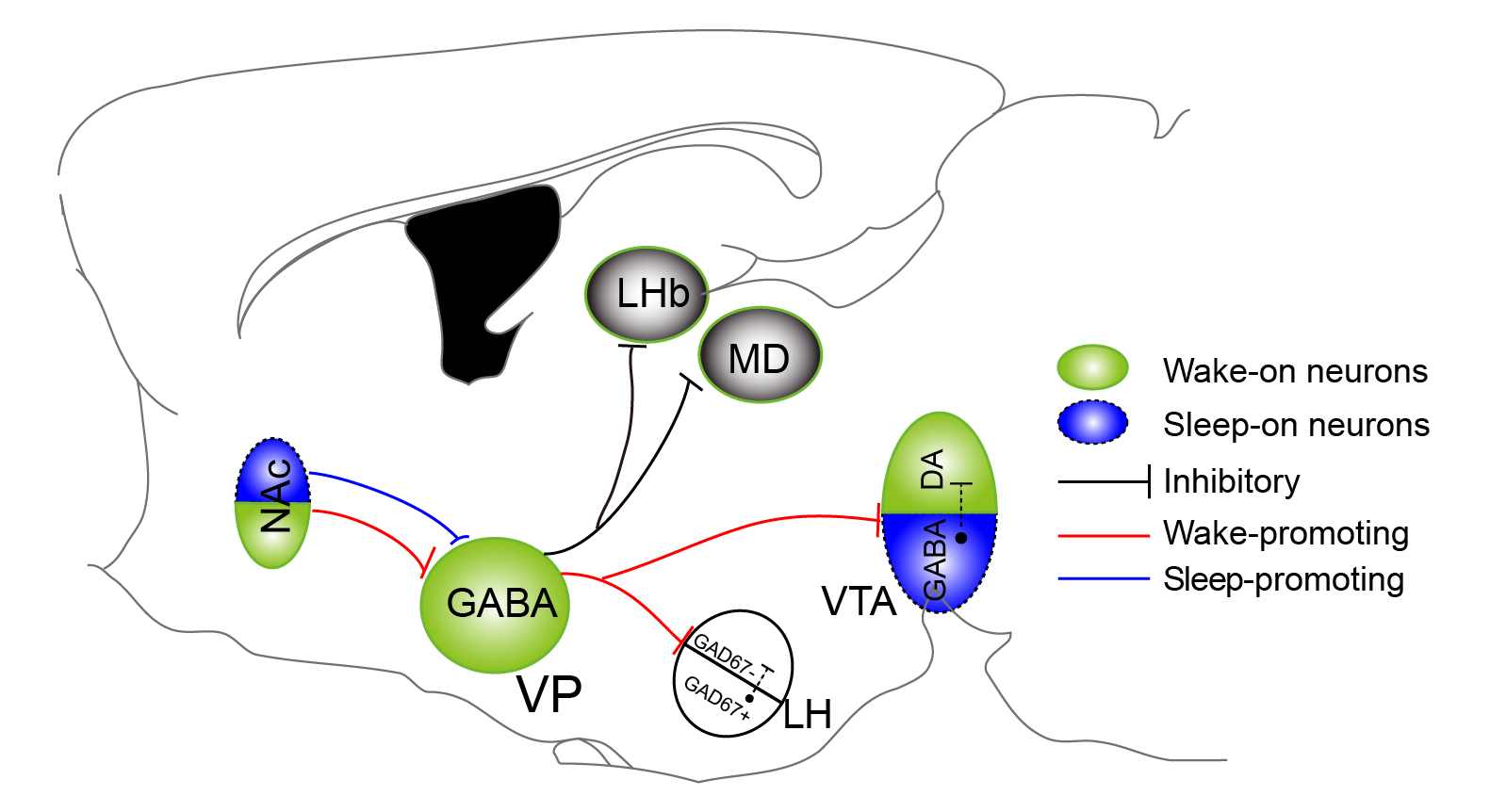


**SI Fig. 10 Proposed circuitry underlying the role of the ventral basal ganglia in sleep-wake regulation.**

In the ventral basal ganglia, NAc wake-on (D_1_R) neurons and sleep-on (D_2_R) neurons project to the VP to mediate arousal and sleep, respectively. VP GABAergic neurons generally promote arousal through the VTA and LH. Although VP GABAergic neurons inhibit both arousal-reducing GABAergic neurons and wake-promoting dopaminergic neurons in the VTA, the VP^GABA^-VTA projection promotes arousal. The VP^GABA^-LH pathway also regulates wakefulness, partially through disinhibition of orexin neurons. VP^GABA^-MD and VP^GABA^-LHb circuits are not involved in sleep-wake regulation. Some subtypes of GABAergic interneurons (e.g., somatostatin neurons) in the VP may promote sleep by inhibiting VTA- or LH-projecting neurons and receive projections from NAc D_1_R neurons. Taken together, VP GABAergic neurons play a key role in integrating information in the ventral basal ganglia and modulate arousal.
